# A multidisciplinary approach to a unique palaeolithic human ichnological record from Italy (Bàsura Cave)

**DOI:** 10.7554/eLife.45204

**Published:** 2019-05-14

**Authors:** Marco Romano, Paolo Citton, Isabella Salvador, Daniele Arobba, Ivano Rellini, Marco Firpo, Fabio Negrino, Marta Zunino, Elisabetta Starnini, Marco Avanzini

**Affiliations:** 1 Evolutionary Studies Institute (ESI), School of GeosciencesUniversity of the WitwatersrandJohannesburgSouth Africa; 2 CONICET-Consejo Nacional de Investigaciones Científicas y TécnicasBuenos AiresArgentina; 3 MUSE, Museo delle ScienzeTrentoItaly; 4 Museo Archeologico del FinaleFinale Ligure BorgoItaly; 5 Dipartimento di Scienze della Terra, dell’Ambiente e della Vita (DISTAV)Università degli Studi di GenovaGenoaItaly; 6 Dipartimento di Antichità, Filosofia, Storia (DAFIST)Università degli Studi di GenovaGenoaItaly; 7 Grotte di ToiranoToiranoItaly; 8 Soprintendenza Archeologia Belle Arti e Paesaggio per la Città Metropolitana di Genova e le province di Imperia, La Spezia e SavonaGenoaItaly; 9 Dipartimento di Civiltà e Forme del SapereUniversità di PisaPiseItaly; Max Planck Institute for Chemical EcologyGermany; Yale UniversityUnited States

**Keywords:** human, bear, dog, None

## Abstract

Based on the integration of laser scans, sedimentology, geochemistry, archeobotany, geometric morphometrics and photogrammetry, here we present evidence testifying that a Palaeolithic group of people explored a deep cave in northern Italy about 14 ky cal. BP. Ichnological data enable us to shed light on individual and group level behavior, social relationship, and mode of exploration of the uneven terrain. Five individuals, two adults, an adolescent and two children, entered the cave barefoot and illuminated the way with a bunch of wooden sticks. Traces of crawling locomotion are documented for the first time in the global human ichnological record. Anatomical details recognizable in the crawling traces show that no clothing was present between limbs and the trampled sediments. Our study demonstrates that very young children (the youngest about 3 years old) were active members of the Upper Palaeolithic populations, even in apparently dangerous and social activities.

## eLife digest

The fossil traces of Stone Age humans and other animals in the Grotta della Bàsura cave system in Italy have been studied since the 1950s. Italian archaeologist Virginia Chiappella published the first studies; she documented bones from an extinct cave bear, human and animal footprints, charcoal from torches, finger marks, and lumps of clay stuck on the walls. Since then, many more archeologists and anthropologists have studied the cave and its fossils. Yet there are still lessons to be learned from this prehistoric site.

Now, Romano et al. have combined a number of different approaches and used some of the latest technology and cutting-edge software to analyze 180 footprints and other tracks found in the cave. These trace fossils date to about 14,000 years ago, and the analysis revealed that they were left by a group of Stone Age humans who descended at least 400 meters into the cave. The group consisted of two adults, an adolescent and two children of about three and six years old. At one point they had to crawl through a low tunnel – something that has not previously been documented in the fossil record. The group were all barefoot, had no clothing on their arms and legs and used wooden torches to light the way.

Together, these findings suggest that young children were active group members during the late Stone Age, even when carrying out apparently dangerous activities. Romano et al. now hope that their multidisciplinary approach may help other scientists looking to understand how humans behaved elsewhere in the world at various points in history.

## Introduction

**Figure 1.**
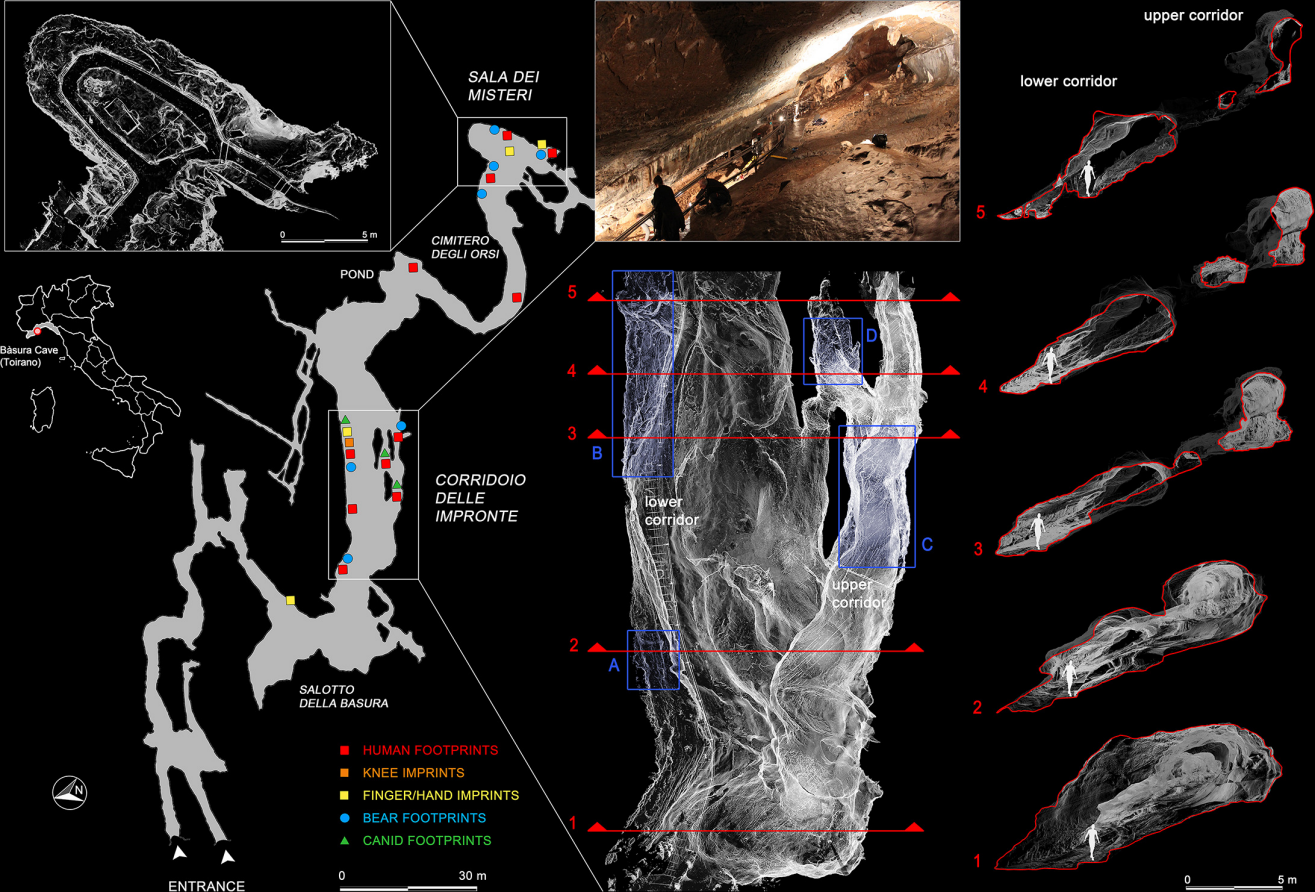
Planimetry of the ‘*Grotta della Bàsura’* and location of human, bear and canid footprints. White rectangles enclose the three-dimensional reconstructions, obtained via laser scanner, of the innermost room (‘*Sala dei Misteri*’ - left) and the main gallery (*‘Corridoio delle impronte’* - right) of the cave, where the human footprints are preserved. Cross-sections obtained from the three-dimensional reconstruction of the main gallery are highlighted in red and show the branching of the ‘lower’ and ‘upper’ corridors, respectively. Blue rectangle indicate the four areas within the main gallery where most of the human footprints are concentrated (A and B for the lower corridor, C and D for the upper corridor).

Discovered in 1950, the hypogeal part of the ‘Grotta della Bàsura’ is a large and deep cave that has produced some of the most important Italian Palaeolithic discoveries of the twentieth century (Chiappella, 1952; Tongiorgi and Lamboglia, 1954; Blanc, 1960; Lamboglia, 1960; Giacobini, 2008) consisting of traces of human activity, especially footprints, dating back to 14 ky cal. BP. Up till 1890 only the atrial part of the cavity was known where Neolithic and late Roman archaeological finds had been unearthed (Maineri, 1985). The cave opening is situated at 186 m a.s.l. about 1 km north of Toirano (Savona, Italy - 436253.433 E; 4887689.739 N), and extends 890 m into Mount S. Pietro with an elevation difference of +20/−22 m relative to that of the entrance (Figure 1).

The inner rooms became accessible in 1950, after the rupture of a stalagmite column, placed a few dozen meters from the entrance, that prevented any access to the cave (Tongiorgi and Lamboglia, 1954; Blanc, 1960; Lamboglia, 1960). The exceptional prehistoric and paleontological value of the cave was firstly recognized by Virginia Chiappella (Chiappella, 1952) who was the first scholar to visit the site soon after its discovery. Chiappella identified several bones of *Ursus spelaeus* and traces both of animal and human frequentation (footprints, charcoals, digital tracks, lumps of clay adhering to the walls) in different areas of the cave within approximately 350 m of the entrance. Regrettably, destruction of most of the ichnological record occurred as a result of uncontrolled cave visits by local villagers and tourists lured to the remarkable discovery as a result of media reports (Blanc et al., 1960; De Lumley and Giacobini, 1985).

The first study of human footprints from the Cave of Bàsura was conducted by Pales (1960), based on original images and 13 plaster casts of the best-preserved specimens from various sectors of the cave. Analysis of the bone architecture of the footprint makers and the apparent relationship with remains of *Ursus spelaeus*, led the author to consider that the footprints were made by ‘Neanderthal-type’ producers. Subsequent analysis (de Lumley and Vicino, 1984), accompanied by radiometric dating, placed the prehistoric visitation of the Bàsura in the Upper Palaeolithic, between 12,000 and 14,000 years uncal BP (de Lumley and Vicino, 1984; De Lumley and Giacobini, 1985). Dating of charred wood fragments found on the trampled surface provided a more precise age for cave visitation at 12,340 years ± 160 years BP (Molleson et al., 1972; Molleson, 1985).

Based on the ichnological study of Pales, Blanc proposed that the inner room called ‘Sala dei Misteri’ was reached by multiple individuals, including a juvenile. Blanc (1960) described, from the same room, a group of seven human footprints identified by heel tracks positioned only a few centimeters from the main wall of the chamber to which numerous lumps of clay were attached. The close association of the footprints with lumps of clay resulted in it being considered the result of initiation rites involving young hunters. This hypothesis was supported by the presence of a stalagmite concretion (defined by Blanc as ‘*acephalous sphinx*’ or ‘*zoomorphic stalagmite’*) placed against the terminal wall of the ‘Sala dei Misteri’ (Mysteries’ Hall) chamber on which several sinuous furrows had been made intentionally by several individuals using their fingers. This interpretation is currently under review by two of the present authors (ES, MZ). The aim of this work is to review and improve understanding of the Grotta della Bàsura’s human ichnology, soil micromorphology, sedimentology and radiocarbon chronology. A preliminary report from this study focused on human footprints preserved in the innermost chamber of the cave (Citton et al., 2017).

In the present paper, we expanded our study to analyze and interpret all human traces (footprints and handprints as well as other traces) from the ‘Grotta della Bàsura’, providing new insight on the behavior, identity of the members, their exploratory techniques, and the social structure of an Upper Palaeolithic group.

### Data

**Figure 2.**
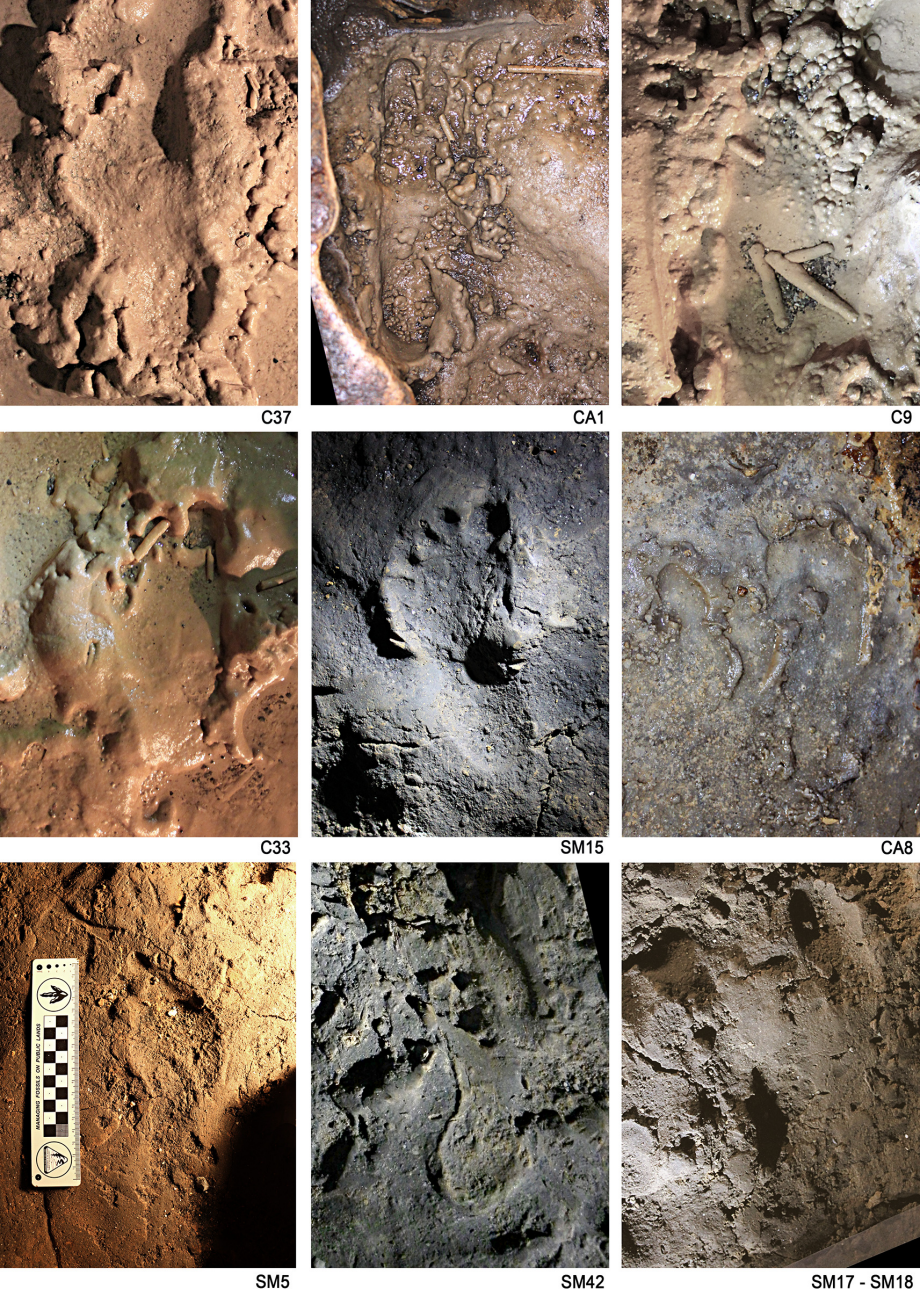
Human footprints imprinted on muddy substrate in different moisture conditions. C37, Human footprint referred to the Morph. 5 (‘lower corridor’). CA1 and C9, Human footprint referred to the Morph. 4 (‘upper corridor’). C33, Human footprint referred to the Morph. 3 (‘lower corridor’). SM15, Human footprint referred to the Morph. 3 (‘Sala dei Misteri’). CA8, Human footprint referred to the Morph. 3 (‘upper corridor’). SM5 and SM42, Human footprint referred to the Morph. 2 (‘Sala dei Misteri’). SM17 and SM18, Human footprint referred to the Morph. 1 (‘Sala dei Misteri’).

**Figure 3.**
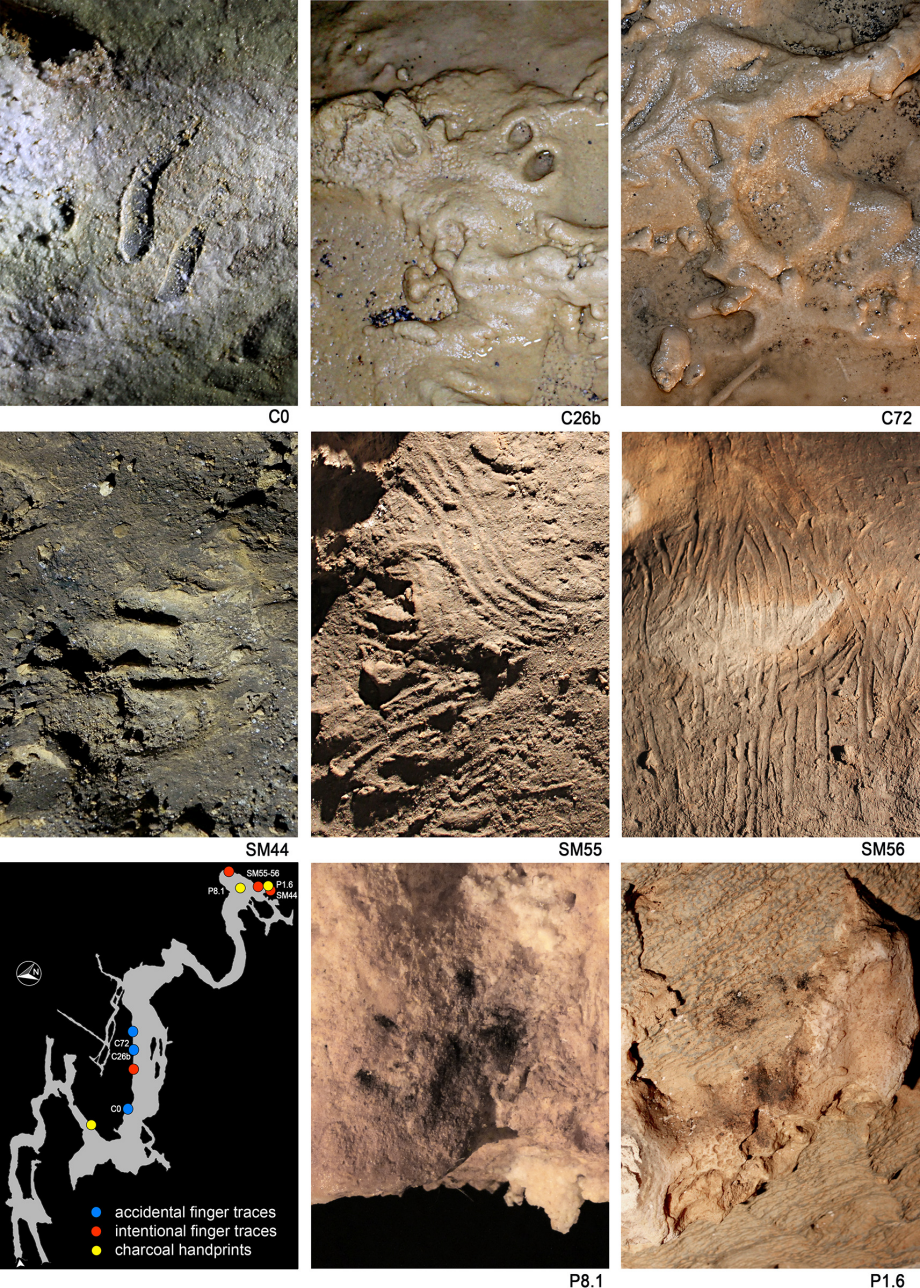
Finger and hand prints. C0, Two finger traces on the concretioned side-wall of the ‘lower corridor’. C26b, Finger traces (‘lower corridor’). C72, Hand print (‘lower corridor’). SM44, finger traces (‘Sala dei Misteri’). SM55, Finger flutings on the clay on the clay floor (‘Sala dei Misteri’). SM56, Finger flutings on a clay-coated stalagmite (‘Sala dei Misteri’). P8.1, P1.6, Coal dirtied handprints (‘Sala dei Misteri’).

A total of 180 footprints and traces *sensu lato* were recorded and studied (Supplementary file 1). In addition to footprints (Figure 2), among the traces are digit and handprints on the clay-rich floor, and smears from hands dirtied with charcoal on the side walls of the cave (Figure 3) (Giannotti, 2008). A paleo-archaeological excavation was performed in 2016 in the ‘*Sala dei Misteri*’. This survey highlighted the total absence of archaeological material but led to the recovery, on the trampled palaeosurface, of numerous charcoal remains with bundles of *Pinus* t. *sylvestris*/*mugo* originally used to illuminate the cave. New radiometric dating on these charcoal samples constrains the exploration of the cave to the late Upper Palaeolithic, between 12,310 ± 60 and 12,370 ± 60 BP, that is from about 14,700 to 14,000 cal BP (Table 1).

**Table 1.** Radiometric dating of charcoals collected from the trampling palaeosurface during 2017 excavations inside the ‘Sala dei misteri’. *14C ages have been calibrated to calendar years with software program: OxCal, version 4.3. Used calibration curve: IntCal13.

Sample name	Provenance	Dated material	Description	Lab. code	F^14^C ± 1σ	^14^C Age (yr BP) ±1σ	Calibrated* dating result (95.4 probability)	%C	%N	δ^13^C (in ‰;±1σ)	δ^15^N (in ‰;±1σ)	C/N ratio
Bàsura SM B5 17B	‘*sala dei misteri’*, square B5, Unit 1	Charcoal (AAA)	*Pinus t. sylvestris/mugo*	GrA-69598	0.2160 ± 0.0016	12310 ± 60	12720–12110 cal BC	71.4	-	‐25.24 ± 0.14	-	-
Bàsura SM D6 33B	‘*Sala dei misteri’*, square D6, Unit 1	charcoal (AAA)	*Pinus t. sylvestris/mugo*	GrA-69597	0.2145 ± 0.0016	12370 ± 60	12830–12165 cal BC	63.3	-	‐26.37 ± 0.14	-	-

**Figure 4.**
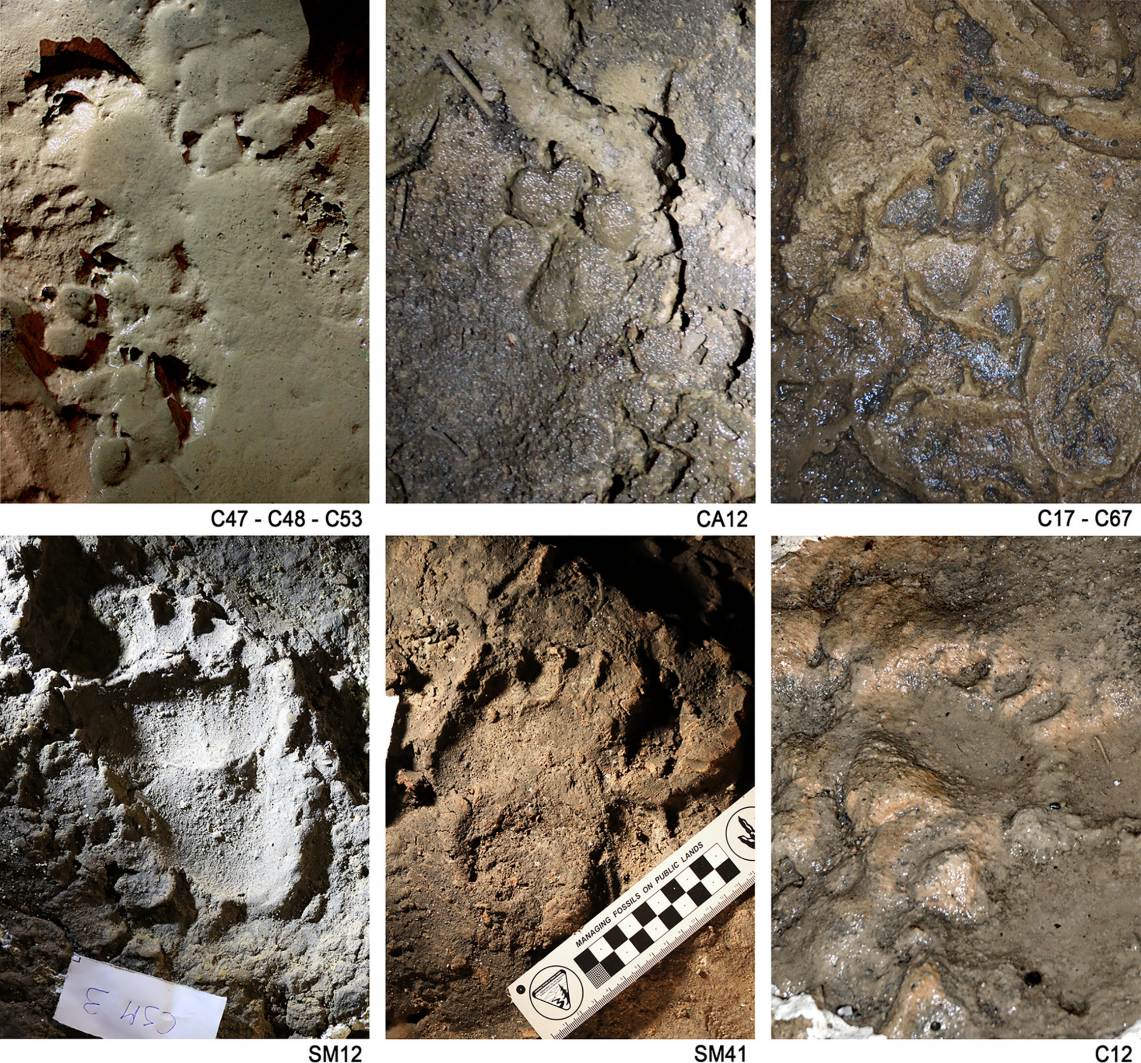
Canidae *incertae sedis* and bear footprints. C47-C48-C53 Canidae footprint on saturated mud (‘upper’ corridor). CA12 well preserved Canidae footprint (‘upper corridor’). SM12-SM41 bear footprint (Sala dei Misteri). C12 bear handprint (‘lower corridor’).

Digit and hand traces are preserved in several sectors of the cave (Figure 3). Most are unintentional traces related to cave exploration activities (Figure 3, C0, C26b, C72). Others, especially in the inner chamber (*‘Sala dei Misteri’*) which are still being studied, are very probably related to social or symbolic activities and can be instead considered intentional (Figure 3, SM55, SM56). Moreover, different sized bear and Canidae *incertae sedis* footprints are ubiquitously present (Figure 4), and are often associated with human prints. The available data regarding the footprints attributed to ‘canids’ suggest a very reduced number of individuals and a close association with the human prints. Should ongoing studies (Avanzini et al in prep.) confirm that the ichnnological association of Bàsura could prove crucial to shed new light on dog domestication in the Upper Paleolithic (Morey and Jeger, 2015; Perri, 2016; Lupo, 2017; Janssens et al., 2018).

*Ursus* sp. hibernation areas are still recognizable with well-preserved nests of both cubs and adult bears.

### Preservation

Footprints are preserved in several areas of the cave, particularly in the innermost chamber (‘*Sala dei Misteri*’) and in the main gallery (*‘Corridoio delle impronte’ – Footprints Corridor*), which is divided into two corridors at different elevations of about 5 m (referred to as the lower and upper corridor, respectively) (Figure 1).

Flooding dynamics and cave geometry produced two different situations for sediment deposition and transport inside the cave. Detrital sediment comprising silty clay and well-sorted sandy sediment are most abundant on the floor of the ‘*Sala dei Misteri*’. Coarse lithologies comprising gravel-sized and larger (>2 mm) grains include a few fragments of bear bones. The sandy fraction comprises allogenic, surface-derived siliciclastic sediment. The ‘*Sala dei Misteri*’ appears to have undergone episodic filling and erosion as a result of catastrophic storms.

Sediment in the *‘Corridoio delle impronte’* comprises a large mud fraction and includes many coarse lithic fragments which are mainly carbonates (calcite and dolomite), suggesting an autogenic origin. Here, the trampled substrate is poorly consolidated and superimposed on a stalagmite crust. At the time when humans and other large mammals left their traces the cave substrate differed in different areas of the cave. In some areas, the substrate was plastic and in other areas it was waterlogged or submerged. Differing moisture content of the substrate accounts for the variable preservation of detail of the tracks (e.g. registration of plantar arch, heel and metatarsal regions, digit tips, track walls), particularly of the associated extra-morphologies (e.g. expulsion rims, slipping traces). The surface of the substrate and the footprints are cross-cut by mud cracks, suggesting a loss of moisture in sediments after trampling. Carbonate crusts (comprising both calcite and dolomite) cover many of the footprints in areas subjected to more intense dripping. Iron and manganese oxide coatings were found in the crust, probably due to repeated immersion in ponded water.

## Results

**Figure 5.**
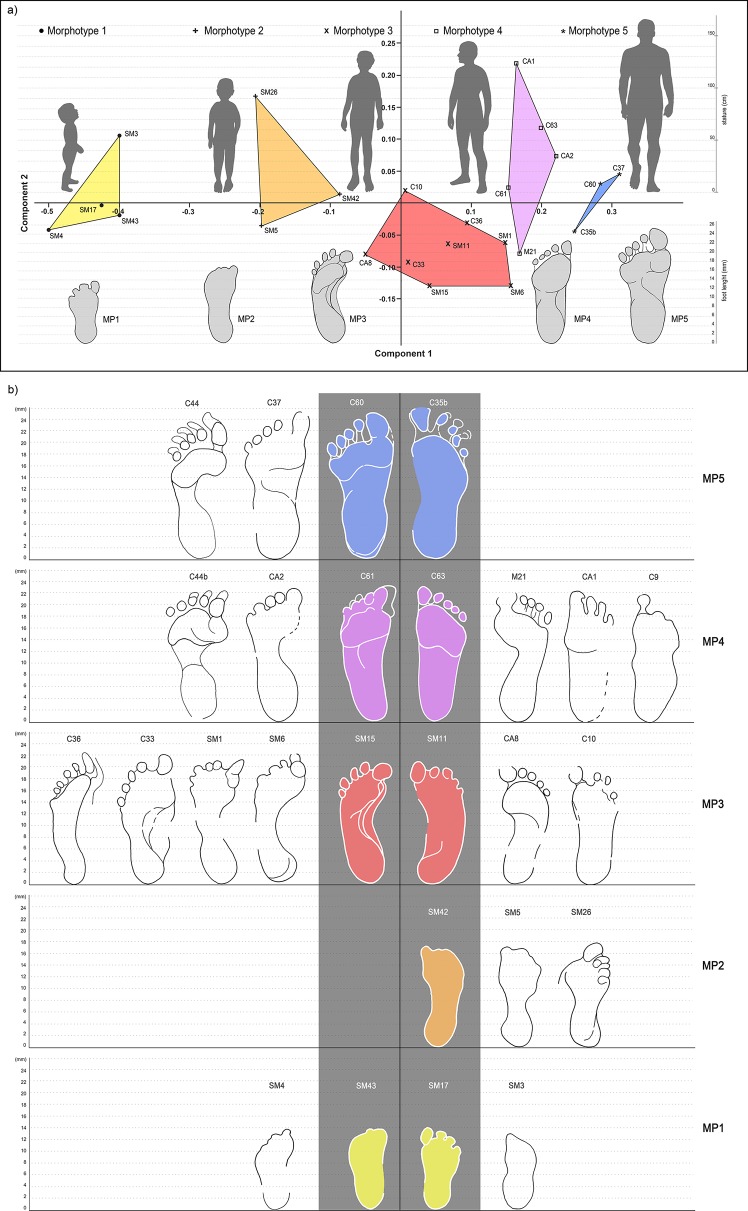
Principal Component Analysis based on the best-preserved footprints from *‘Sala dei Misteri’* and *‘Corridoio delle impronte’*. (**a**) The five morphotypes to which footprints have been referred are shown above. (**b**) Selected outlines of the best preserved footprints, for each recognized morphotype, are reported.

Geometric morphometry performed on human footprints highlighted five main morphotypes (hereafter Morphs.) indicating a possible minimum number of five individuals entering the cave (Figure 5). This number is confirmed by the construction of morphological groups (Table 2) reconstructed through the overlapping of footprints that show a variability of less than 2% of the main parameters.

**Figure 5—figure supplement 1.**
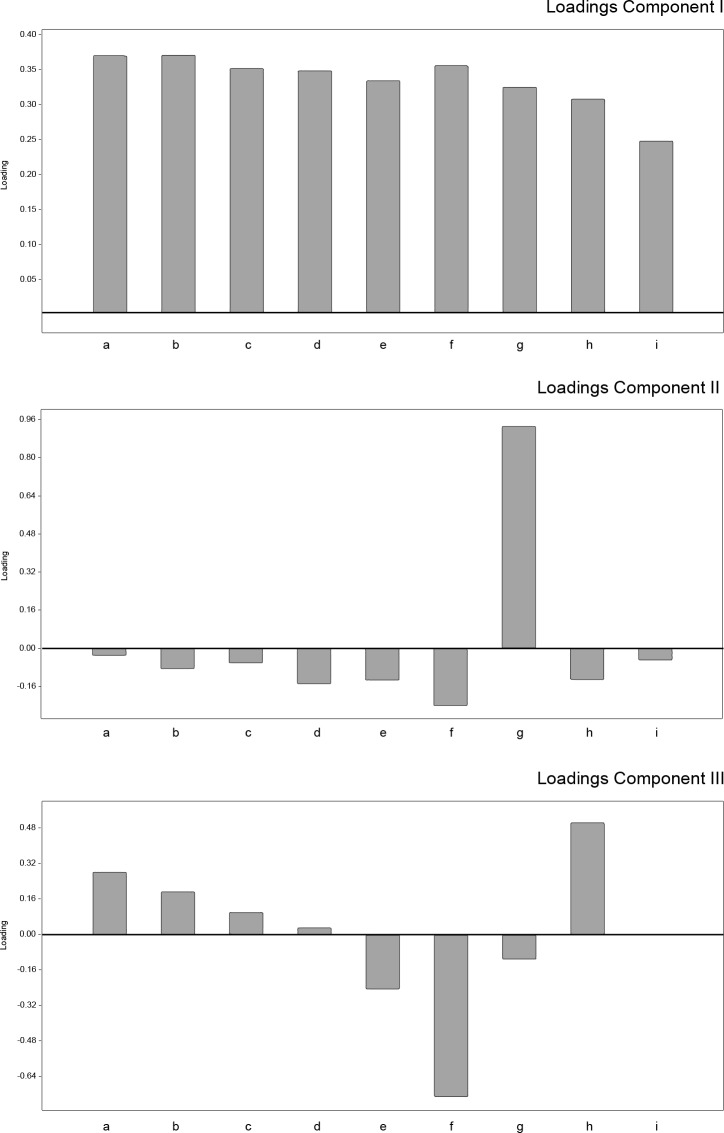
Loadings for the first three principal components. (**a**) Dt1-BL; (**b**) Dt2-BL; (**c**) Dt3-BL; (**d**) Ball medial (mtm-BL); (**e**) Ball lateral (mtl-BL); (**f**) Heel medial (ccm-BL); (**g**) Heel lateral (ctul-BL); (**h**) Ball (mtm-horiz); (**i**) Heel (ctul-horiz). Anatomical abbreviations as in Methods section.

**Table 2.** Footprint shape features after **‘**Robbins footprint recording form**’** (1985, p.97–102).

ID	Left (L) or right (R) foot	General appearance		Relative length of toes	Toes region general appearance length - width	Toe one position	Ball region, general appearance length-width	Arch region		Heel region, general appearance	Hell posterior margin	
		Length	Width					Medial margin	Lateral margin			
SM3	R	short	broad	1, 2, ?	short - broad	extended, anteriorly		straight	concave	circular	convex pronounced	Morphotype 1
SM4	L			1,2,3,4,5	short - broad	extended, anteriorly	short - moderate		concave			
SM43	L	short	broad	1,2, ?	short - broad	extended, anteriorly		straight	convex		convex pronounced	
SM17	R	short	broad	1,2,3,4,5	short - broad	extended, anteriorly		straight/concave	convex		convex pronounced	
SM5	R	moderate	moderate	1,2,3,4,5	short - broad	extended oblique medially	long - moderate	concave	concave		convex slight	Morphotype 2
SM42	R	moderate		1,2, ?		extended oblique medially		concave			convex pronounced	
SM26	L	moderate	broad	1,2,3,4,5	short - moderate	extended oblique laterally		concave	concave		convex slight	
CA8	R	long	moderate	1,2,3,4,5	moderate - broad	extended oblique medially	long - moderate	concave	straight	oblong	convex pronounced	Morphotype 3
CA10	R	long	moderate	1,2,3,4	moderate - broad	extended oblique medially		straight/concave	straight	oblong	convex pronounced	
SM15	L	long	moderate	2,3,1,4,5	moderate - broad	extended oblique medially	long - narrow	concave	straight	oblong	convex pronounced	
SM11	R	long	moderate	1,2,3,4,5	short - broad	extended anteriorly		straight/concave	convex	oblong	convex pronounced	
SM6	L	long	moderate	2,1,3,4,5	short - broad	flexed slight	long - narrow	concave	convex	oblong	convex pronounced	
SM1	L	long	moderate	2,3,1,4,5	short - broad	extended oblique medially	moderate - narrow	unknown	convex	circular	convex pronounced	
C33	L	long	broad	1,2,3,4,5	long - broad	extended oblique medially	long - broad	concave	convex	oblong	convex pronounced	
C36	L	long	very narrow	1,2,3,4,5	moderate - narrow	extended oblique laterally	long - narrow	concave	straight	oblong	convex pronounced	
CA1	R	long		1,2,3,4	moderate - broad	extended anteriorly	moderate - narrow	straight		oblong	convex, pronunced	Morphotype 4
CA2	L	long	moderate	1,2,3,4,5	moderate - broad	extended anteriorly	moderate - narrow	concave	straight	oblong	convex pronunced	
C61	L	long	moderate	1,2,3,4,5	moderate - broad	extended oblique medially	moderate - narrow	straight/concave	straight/convex	oblong	convex pronunced	
C63	R	long	moderate	1,2,3,4,5	moderate - broad	extended anteriorly	moderate - narrow	straight/concave	straight/concave	oblong	convex pronunced	
M21	R	long	moderate	1,2,3,4,5	moderate - broad	extended anteriorly	moderate - narrow	concave	straight	oblong	convex pronunced	
C9	R	long	moderate	1		extended anteriorly	moderate - narrow	straight	convex	oblong	convex pronunced	
C44b	L	long	broad	2,1,3,4,5	short - broad	extended oblique medially	moderate - broad	concave	convex	oblong	convex, moderate	
C60	L	long	broad	1,2,3,4,5	moderate - broad	extended oblique laterally	moderate - broad	straight/concave	straight/concave	circular	convex moderate	Morphotype 5
C37	L	long	broad	1,2,3,4,5	moderate - broad	extended anteriorly	moderate - broad	straight/concave	straight/concave	circular	convex moderate	
C35b	R	long	broad	1,2,3,4,5	moderate - broad	extended anteriorly	moderate - broad		straight/concave	circular	convex moderate	
C44	L	long	broad	1,2,3,4,5	moderate - broad	extended anteriorly	moderate - broad	concave	straight/concave	oblong	convex pronunced	

Morphs. 1 and 2 can be easily distinguished on the basis of the absolute footprint size. Morph. 1 includes footprints with a length of 13.55 ± 0.49 cm showing characters indicative of an early ontogenetic stage of the producer, such as digit traces and the heel area proportionally wider than those of the longer tracks. Morph. 2, with a length of 17 cm, is distinguished from Morph. one on the basis of a more pronounced plantar arch. Morph. 3 comprises footprints 20.83 ± 0.51 cm in length (Figure 5 and Table 3). The plantar area is characterized by a very pronounced medial embayment. This corresponds with a strongly convex external margin, as is shared together with a strong adduction of digit I trace, an overall larger divarication of digit traces and a consistent separation between adjacent digits II-III and IV-V.

**Table 3.** Measurements and elaboration data (foot index, stature, body mass and age) based on the best-preserved tracks from the ‘Sala dei Misteri’ and ‘Corridoio delle impronte’. *Body mass: (a) Citton et al., 2017; (b) Bavdekar et al., 2006; (c) Grivas et al., 2008 (see text).

		LENGTHS											WIDTHS			ANGLES
		FOOT					BALL		ARCH		HEEL		BALL	ARCH	HEEL	Angle of toe declination
ID	Left (L) or right (R) foot	Dt1 (cm	Dt2(cm)	Dt3(cm)	Dt4(cm)	Dt5(cm)	medial (mtm-BL)(cm)	lateral (mtl-BL)(cm)	medial (ntu-BL)(cm)	lateral (mttu-BL)(cm)	medial (ccm-BL)(cm)	lateral (ctul-BL)(cm)	mtm- horiz(cm)	mttu-horiz(cm)	ctul-horiz(cm)	T1-T5(degrees)	max FL (cm)	max FW (mtm-mtl) (cm)	arc angle(degrees)	Foot index	stature(cm)	body mass *(kg)	age	
SM3	R	13	12.3				10.5	9.2	5.5	6.8	2.3	2.4	5.5	4.7	4.3		13	6	20	0.46	84.36	11.78^(a)^		Morpho type 1
SM4	L	13.5	12.5	11.5	11	9,8	10.2	8	4	5.5	2.2	1.6	5.5	4	4.2	40	13.5	6.5	22	0.48	87.61	12.55^(a)^		
SM43	L	13.5	13.5				10.5	9.4	5.5	6.5	2.2	1.8	6	5.5	4.6		13.5	6.5	20	0.48	87.61	12.55^(a)^		
SM17	R	14.2	13.8	13.5	12.6	10.8	10.2	8.5	5	6	2	1.8	6.5	4.8	4	32	14.2	6.8	25	0.48	92.15	13.70^(a)^		
																	13.55 ± 0.49			0.48 ± 0.01	87.93 ± 3,20	12.64 ± 0,79^(a)^	<3	
SM5	R	17	16.8	15.5	14	13	12	12			2,5	2	6.5		5	35	17	6.8	28	0.40	110.32	19.50^(a)^		Morpho type 2
SM42	R	17	16.8				12.5	11.5		10	3.4	2.5	7		5.4		17	7.2	25	0.42	110.32	19.50^(a)^		
SM26	R	18	16.5	15	14						2	3			5		18		28	-	116.81	22.12^(a)^		
																	**17**			0.41 ± 0,02	110.32	19.5^(a)^	5–6	
CA8	R	20.2	19.8	19.4	18.3	16.8	15	13	7.5	9.2	2.6	2	7.5	6	4.5	30	20.2	8	-	0.40	131.08	29.18^(a)^	8–10 boy/9–11 girl	Morpho type 3
C10	R	20.5		19.2	18.4	16.5	15.5	12.5	6.5	8.5	2.8	2.6	7	5.5	5.6	32	20.5	8	-	0.39	133.03	30.30^(a)^		
SM15	L	20.5	20.5	18.5	17.5	16	15.4	13	6.5	7.8	4	2	7	4	6	30	20.5	7	45	0.34	133.03	30.30^(a)^		
SM11	R	21	20.2	19.7	18	16.8	15.4	14.5	5.5	8	3.3	2.3	7.5	6.8	6	30	21	7.5	40	0.36	136.28	32.28^(a)^		
SM6	L	21	21.5	20.5	18.8	17	17.5	15.5	6.8	8.5	4	2.2	9.2	5.5	5.7	20	21.5	9	40	0.42	139.52	34.38^(a)^		
SM1	L	21.2	21.3	20.8	20.4	18.5	17.4	14.8	6.8	8.7	3.8	2.5	8	5	6	20	21.3	8.5	40	0.40	138.22	33.52^(a)^		
C33	L	22.2	21	19.5	17.3	14.8	15.5	10.7	6	7.8	2.5	2	10	6.5	5.8	45	22.2	10.5	48	0.47	144.06	37.55^(a)^		
C36	L	22.7	21.7	19.5	17.8	16.2		14.5			3	2.5			5.5	48	22.7		-	-	147.31	39.99^(a)^		
																	20.83 ± 0.51			0.38 ± 0.03	135.19 ± 3.33	31.66± 2.05 ^(a)^	8–11	
CA1	R	22.4	21.8	20.8	18.5		14.8	12.8	7		3.1		8				22.4	8.5	-	0.38	145.36	45.48^(b)^ - 46.66^(c)^		Morpho type 4
CA2	L	22.5	22	21	20	18.5	17.3	15.2	6	8.6	3.5	3.5	8.8	6	6.4	30	22.5	8.5	44	0.38	146.01	45.66^(b)^ - 47.19^(c)^		
C61	L	23	21.7	20.8	20	19.5	16.4	14	8.5	9.5	3.8	3	7.5	6.5	5.4	30	23	9	45	0.39	149.25	46.57 ^(b)^ - 49.82^(c)^		
C63	R	23.3	22.2	20.8	19.8	18.7	17	14	7.9	9.5	3.5	3.8	8.5	6.7	5.3	32	23.3	9	42	0.39	151.20	47.12^(b)^ - 51.39 ^(c)^		
M21	R	-	21.6	21.3	20.7	19.2	16.7	14.6	7.8	10.5	3.5	2.4	9.6	4.8	6.2		-	9.8	42	-	-	-		
C9	R	22.5											8		5.5		22.5	8	-	0.36	146.01	45.66^(b)^ - 47.19^(c)^		
C44b	L	21.5	21	20	19.5	18.5	15	13	7	4	2.2	1.5	10	5	5.5	20	21.5	10.5	50	0.49	139.52	43.84^(b)^ - 41.93 ^(c)^		
																	22.80 ± 0.42			0.38 ± 0.01	147.96 ± 2.75	46.21 ± 0.77 ^(b)^ - 48.76 ± 2.23 ^(c)^	>14 - adult	
C60	L	25.3	24.2	22.7	21.4	20	18	14.8	6	8	3.5	3.3	10.5	7.5	6.4	35	25.3	11	52	0.43	164.18	50.76^(b)^		Morph otype 5
C37	L	25.7	23.8	22.5	21	19.5	18.4	14.7	6	7.8	3.7	3.5	10.5	7.5	7	35	25.7	10.5	55	0.41	166.77	51.48^(b)^		
C35b	R	26.2	24.8	22.8	20.7	18.7	17	13.7	5.8	7.2	3.7	2.7	9.5	7	6.7	40	26.2	10.5	-	0.40	170.02	52.39^(b)^		
C44	L	25	23.5	22.5	20.8	19	16.8	14.2	5.5	6.7	2.8	2	9.2	5.7	6	45	25	10	50	0.40	162.23	50.21^(b)^		
																	25.73 ± 0.45			0.41 ± 0.02	166.99 ± 2.93	51.54 ± 0.82 ^(b)^	>14 - adult	

**Figure 6.**
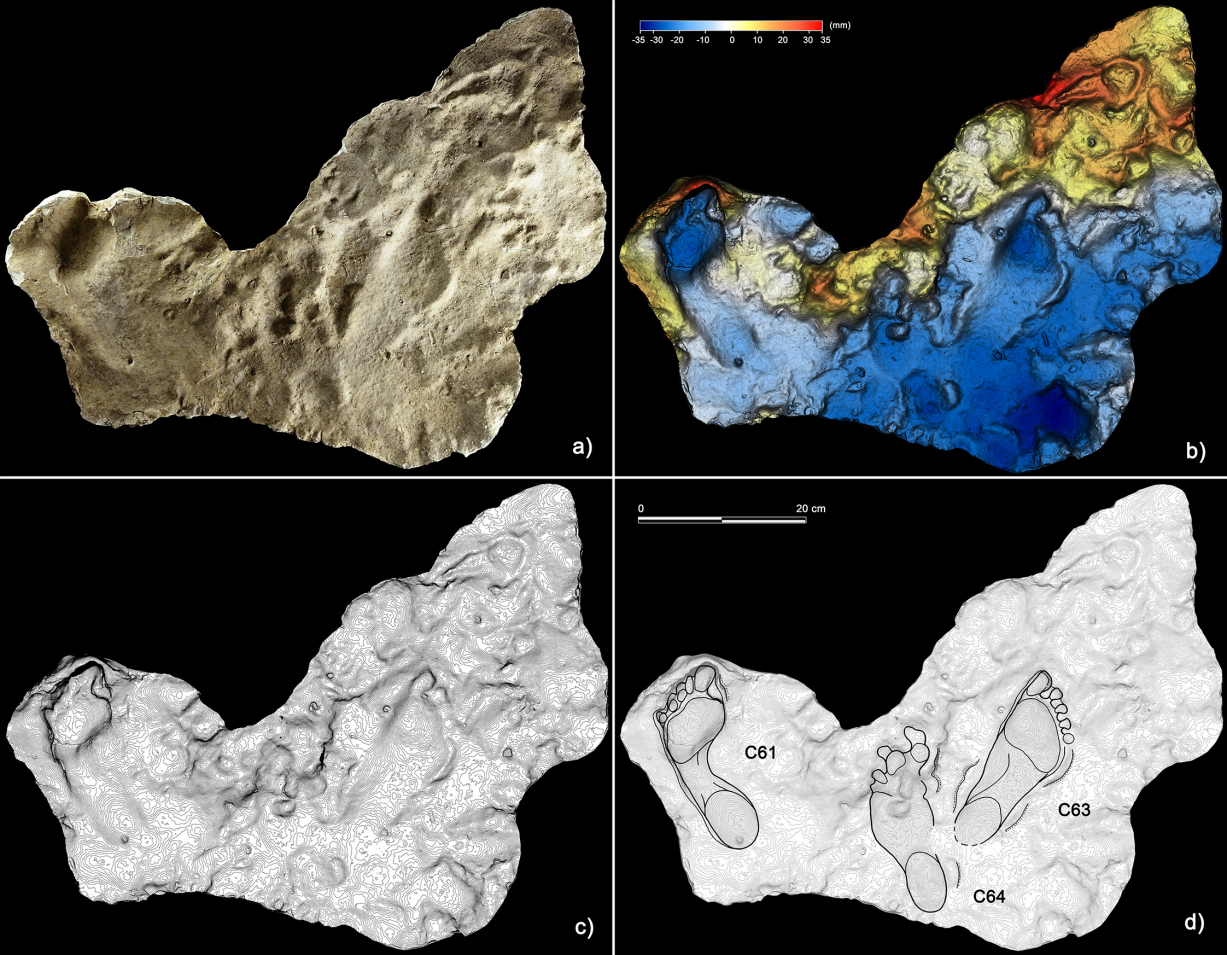
Plantigrade tracks from the ‘lower corridor’. (**a**) Cast of the 1950s reproducing tracks C61, C63 and C64, preserved in the sector A of the ‘lower corridor’ (see Figure 1 main text). (**b**) Digital terrain model of the cast obtained from the HDI 3D Scanner. (**c**) Topographic profile with contour lines, obtained from b. (**d**) Interpretive draw. Note that the tracks C61 and C63 were most likely left by a producer (Morph. 4) crouched against the side-wall of the ‘lower corridor’.

**Figure 7.**
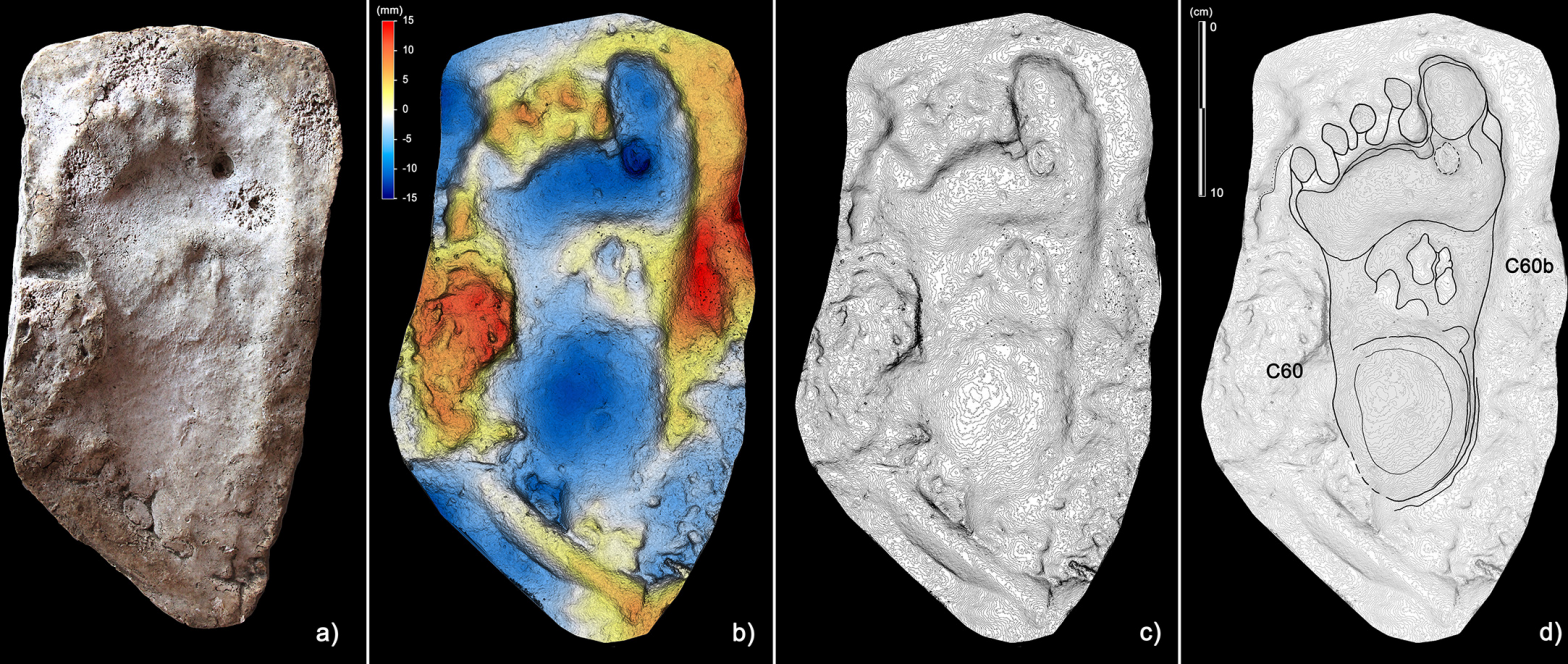
Plantigrade track from the ‘lower corridor’. (**a**) Cast of the 1950s reproducing the track C60 preserved in the sector A of the ‘lower corridor’ (see Figure 1 main text). (**b**) Digital terrain model of the cast obtained from the HDI 3D Scanner. (**c**) Topographic profile with contour lines, obtained from b. (**d**) Interpretive draw. A superimposed partial canid track, C60b, is clearly recognizable in the metatarsal area of the human footprint (Morph. 5).

Morph. 4 (Figures 5 and 6) is represented by larger footprints (22.80 ± 0,42 cm in overall length) with roughly straight medial and lateral margins and a medial embayment less marked than that of Morph. 3. Digit tip traces are strongly aligned and oriented forward. Morph. 5 (Figures 5 and 7) includes footprints of 25.73 ± 0.45 cm in overall length and slightly concave margins, with a variably pronounced plantar embayment. The footprints of Morph. 5 are generally more robust and stockier than those of Morphs. 3 and 4, sharing with Morph. 4 the straight, forwardly oriented digit tips, and with Morph. 3 an adducted digit I trace.

Plantigrade tracks enabled us to estimate stature, weight and ontogenetic stage of the producers based on biometric measurements (Table 3) and the adopted formulas (see Methods). An estimation of the gender for Morph. 5 was also attempted (see Methods).

The group of human track producers entering the cave comprised: a three-year-old child about 88 cm tall (Morph. 1); a child at least 6 years old and about 110 cm tall (Morph. 2); a pre-adolescent, between 8 and 11 years old, about 135 cm tall (Morph. 3); a sub-adult to adult about 148 cm tall (Morph. 4); and an adult about 167 cm tall (Morph. 5). Estimate of the stature for the Morph. 5 is further supported by the length of the tibia derived from the available kneeling traces (see Methods). Our results concerning Morphs. 4 and 5, which are referred to adult individuals, are in agreement with the average stature of European Upper Palaeolithic people (162.4 ± 4.6 cm for males and 153.9 ± 4.3 cm for females) (Villotte et al., 2017).

Body mass estimates derived from footprint parameters suggests slender and muscular body proportions for all the trackmakers. Arch angle and footprint morphology suggests a male as the probable trackmaker of the largest footprint group. Differently, the gender result difficult to infer for morphotypes 1, 2, 3, 4, although Morph. 4 can be referred most probably to a female.

**Figure 8.**
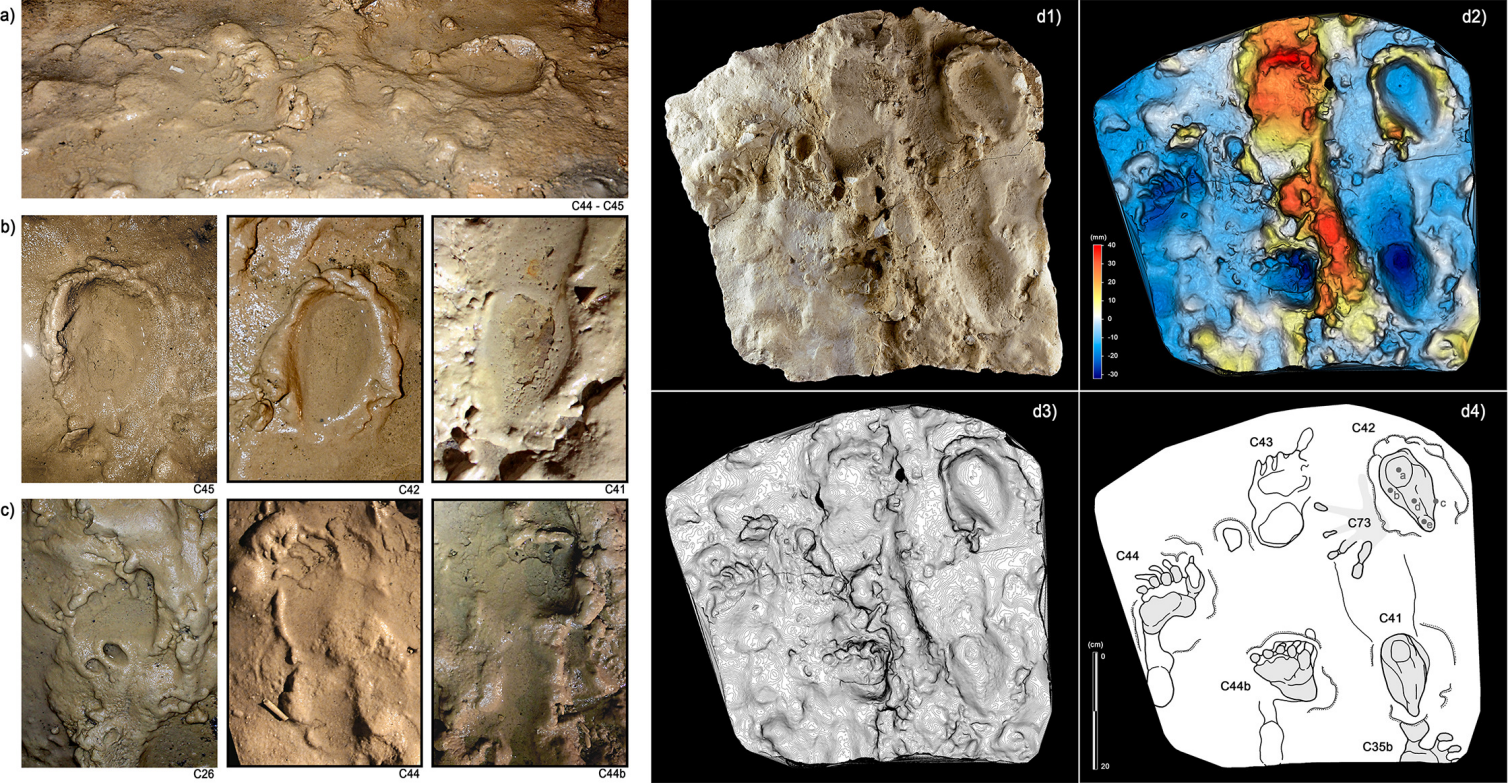
Selection of semi-plantigrade and knee traces from the ‘lower corridor’ of the ‘*Corridoio delle impronte’* in the Bàsura cave, indicating crawling locomotion of the producers. (**a**) Associated metatarsal (C44) and knee (C45) traces allowing estimation of the tibial length of the producer. (**b**) Knee traces (C45, C42 and C41) imprinted on a plastic, waterlogged muddy substrate. (**c**) Metatarsal traces (C26, C44 and C44b) imprinted on a plastic, waterlogged muddy substrate. (d1) cast of the 1950s reproducing two knee (C41 and C42) and two metatarsal (C44, C44b) traces preserved in the area B of the ‘lower corridor’ (see Figure 1). (d2) Digital Terrain Model obtained from the HDI 3D Scanner. (d3) Topographic profile with contour lines, obtained from d2. (d4, Interpretive draw. In the knee trace, C42 are located the impressions of the patella (**a**), vastus medials (**b**), the fibular head (**c**), the patellar ligament (**d**) and the tibial tuberosity (**e**).

Digitigrade and semi-plantigrade footprints provide information on pedal postures and the behavior of the producers passing through different sub-environments of the cave. Both these footprint types were, in most cases, traced back to the same type of producer by comparison with complete footprints indicating complete foot support during locomotion. Some semi-plantigrade footprints (e.g. Figure 8, C44, C44b) show a strongly adducted trace of digit I and an apparent alignment with the other digits, probably because of the intra-rotation movements of the distal portion of the foot during the thrust phase. Footprints included in the Morph. 3 show a peculiar pedal morphology. While the resulting morphology of the digit I trace is explained by walking on a waterlogged substrate, the separation between digit pairs II-III and IV-V suggest an inherited familiar trait or a pathological condition of the producer’s feet. The producer was not incapacitated, showing the greatest mobility in the hypogeal environment.

**Figure 9.**
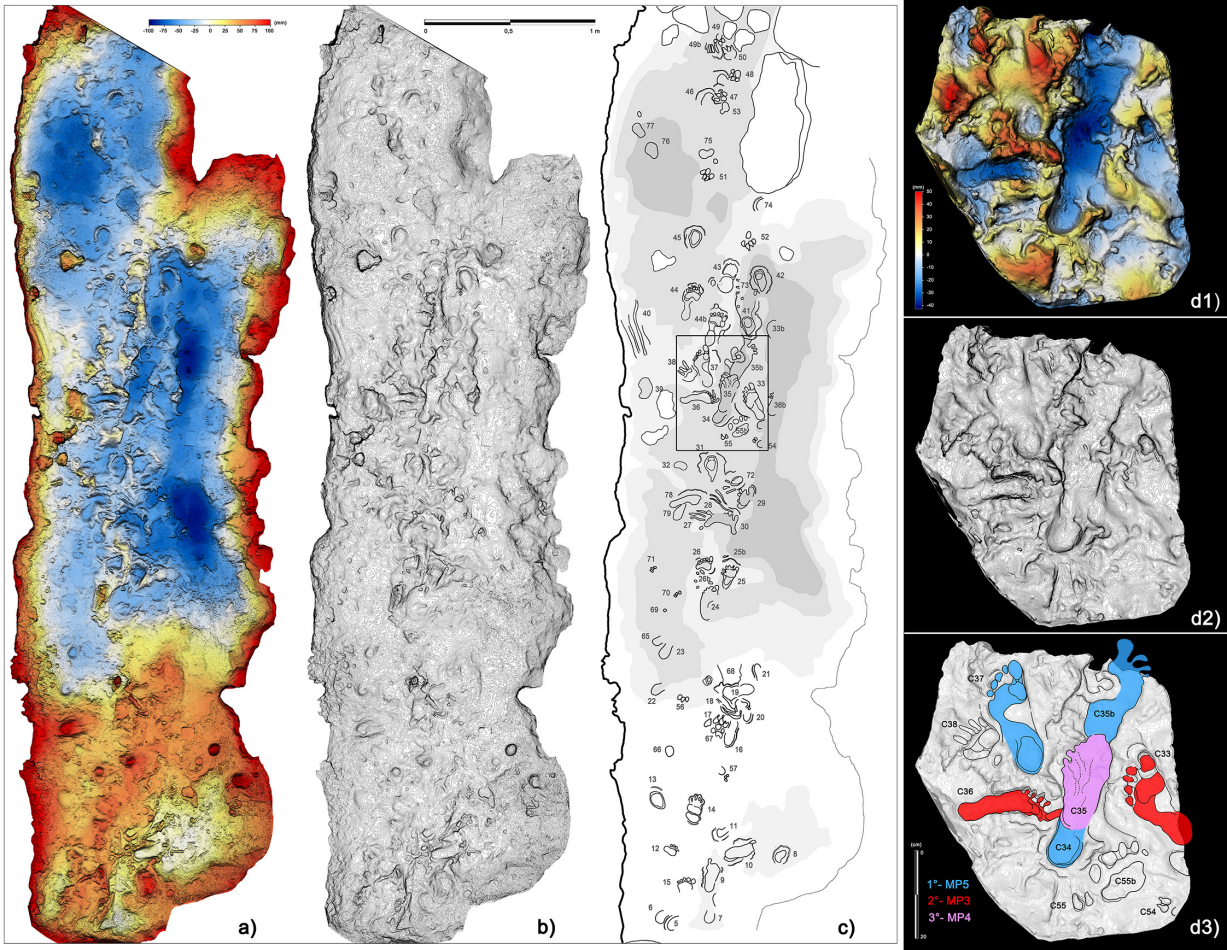
Crawling locomotion in the ‘lower corridor’ (sector B in Figure 1). (**a**) Color topographic profile obtained from the digital photogrammetric model. (**b**) Topographic contoured profile. (**c**) Interpretive draw of the track-bearing surface (numbers identify single tracks and traces and are to be intended as preceded by the letter C). (d1) Digital Terrain Model obtained from a cast of the 1950s reproducing a small area of the ‘lower corridor’. (d2) Topographic profile with contour lines, obtained from d1. (d3) Interpretive draw and timing of the different recognized tracks.

In the lower corridor (Figure 9), a few of these footprints are associated with elongated traces imprinted by the producers’ knees resting on the substrate. Successions of kneeling traces can be clearly recognized for Morphs. 3, 4, and 5. Based on the overall size of the metatarsal and knee couples aligned on the substrate, crawling in a totally unknown environment is inferred for the whole group. These knee imprints (e.g. Figure 8, C42) show the muscle structure of the knee joint and adjacent regions. The patella, the patellar ligament (tendon), the tibial tuberosity, the fibular head, the basis of the vastus medial and the iliotibial band are recognizable and allow us to infer the body structure of the trackmakers.

## Discussion

Successions of kneeling traces allowing one to infer a crawling locomotion for the trackmakers have never been reported before. Isolated kneeling traces (i.e. a footprint followed by a knee imprint of the same leg) have only previously been reported from the ‘Galerie Wahl’ at Fontanet cave and from the ‘Salle des Talons’ at Tuc d’Audoubert cave, France (Pastoors et al., 2015) but are not sufficient to infer crawling. In addition, the anatomic details clearly recognizable in the crawling traces from ‘Grotta della Bàsura’ enabled us to hypothesize that no clothing was interposed between the limb and the trampled sediments.

The integration of all available ichnological evidence with data on the complex morphology of the cave enabled us to reconstruct a detailed hypothesis for the events which took place by Paleolithic peoples while exploring the cave about 14,000 BP.

**Figure 10.**
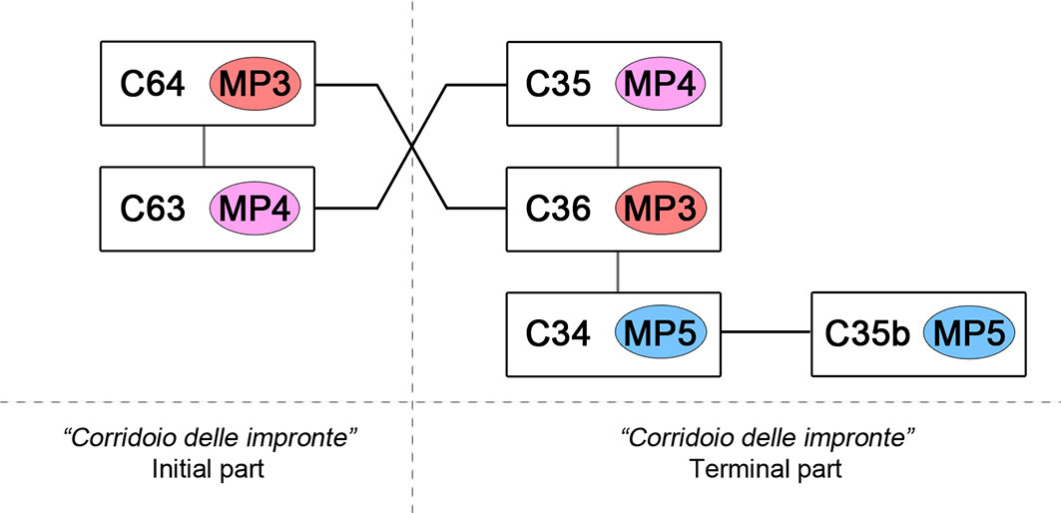
Timing of impressions of human footprints. The interference between footprints attributed to different individuals suggests a single exploring event of the cave. In particular the cross-overlapping of MP3 and MP4 trackmakers confirms their contemporary entry into the main gallery.

The short radiometric interval, documented by both previous radiocarbon dates and those derived from the latest research presented here (Table 1), together with interferences relating to the interrelations of the different footprints, suggest that all individuals entered the cave at the same time. In particular, the group of footprints C34, C35b, C36, C35 in the terminal part of the *‘Corridoio delle impronte’* provides relative timing of the impressions: C35 (Morph. 4) is superimposed on C36 (Morph. 3) and on C34 and C35b (both Morph. 5) (Figure 9, d3). In this specific case, the producer of Morph. 4 passed after the producers of Morphs 3 and 5, respectively. In the entrance of the same corridor, the timing is reversed: footprint C63 (Morph. 4) is superimposed by C64 (Morph. 3) proving that Morphs. 3 and 4 were made at the same time (Figure 10).

It is also difficult to assume that Morphs. 1 and 2 may have entered the deepest part of the cave without the presence of some older individual: if the older individuals who were leading the exploration were either Morphs. 3 or 4 the group consequently increases to include at least four individuals. No relationship can be established for the larger individual; however, as reported below, the fact that the footprints of this individual are regular and were closely followed on the same path by all the other individuals, suggests that the larger one was likely the leader of the synchronous exploration. Thus, all the available lines of evidence, particularly the complex interrelationship of the studied footprints, strongly suggest that a single exploration event by a heterogeneous group of five individuals is the most parsimonious and best supported hypothesis.

Five individuals, comprising two adults, an adolescent and two children, entered the cave barefoot and with a set of *Pinus* t. *sylvestris*/*mugo* bundles which were burned for illumination purposes. The adopted lighting system of several sticks enabled a longer period of lighting, as inferred from the fire wood illumination bundles adopted by the Bronze age salt miners at Hallstatt (Grabner et al., 2007; Grabner et al., 2010). Lighting bundles were usually made of resinous wood (Scots pine or Mountain pine), and called torch wood (Ast, 2001; Théry-Parisot et al., 2018). This interpretation fits with the archaeological evidence from the Bàsura Cave.

**Figure 11.**
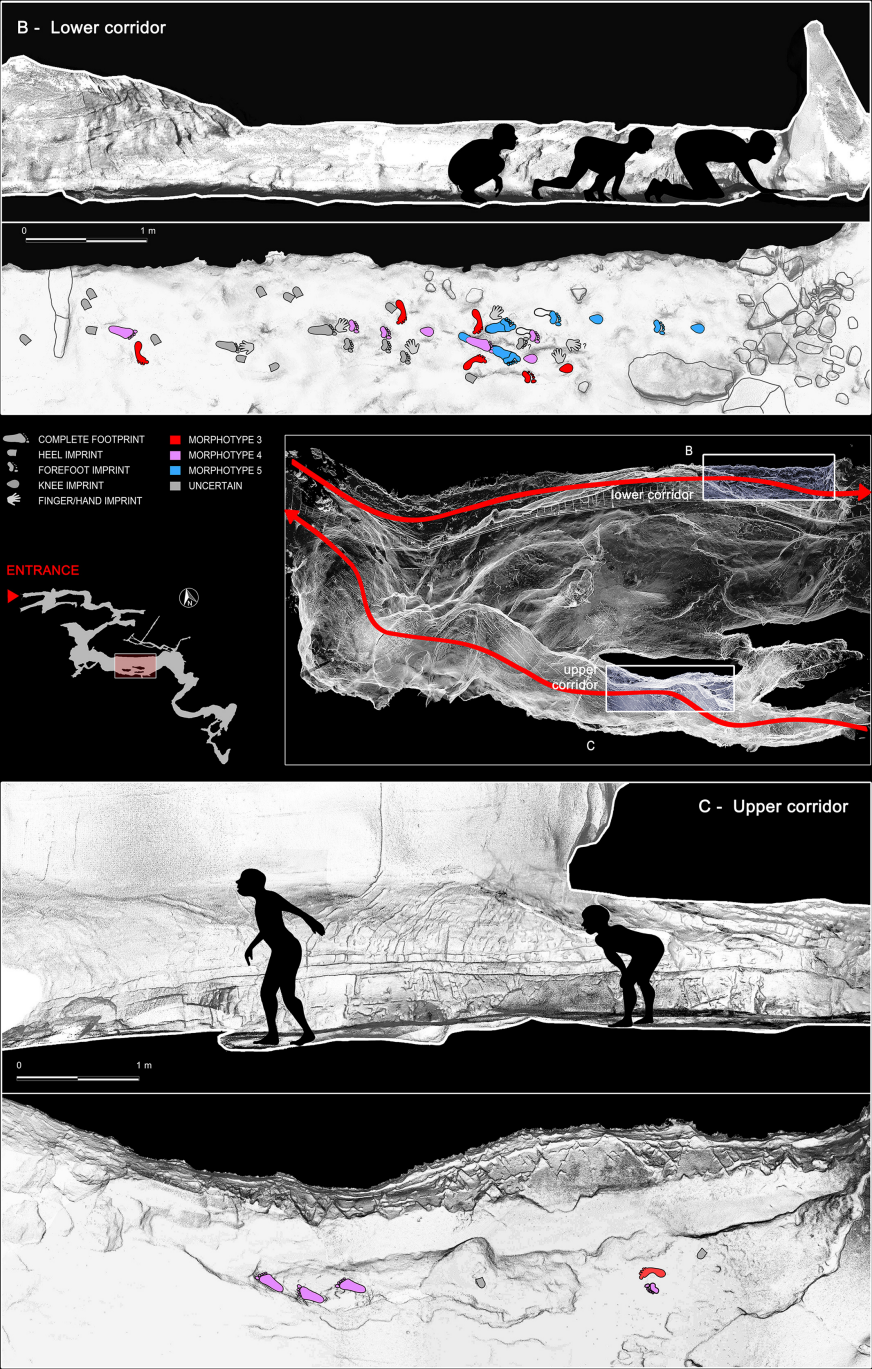
Reconstruction of the exploration routes chosen by the producers to enter and exit the cave. (**B**) Crawling locomotion adopted by the producers to cross the ‘lower corridor’ and access to the innermost rooms of the cave. (**C**) Exit route passing through the ‘upper corridor’, traveled by the producers in complete erect walking. The smallest producers are not reported in the sketch.

**Figure 12.**
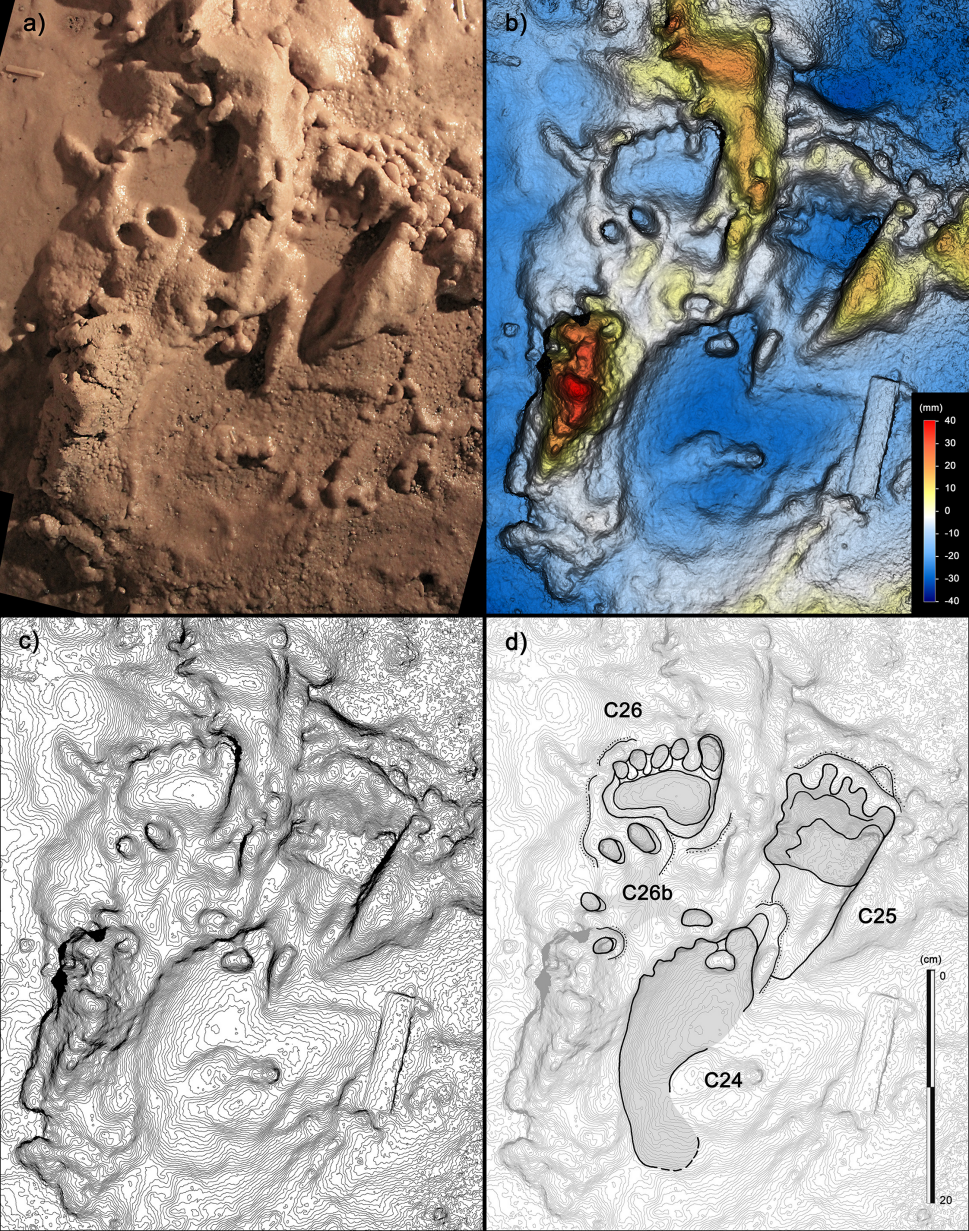
Human tracks from the ‘lower corridor’. (**a**) Tracks C26, C26b, C25 and C24 from the sector B of the ‘lower corridor’ (see Figure 1 main text). (**b**) Digital terrain model obtained from high-resolution photogrammetry. (**c**) Topographic profile with contour lines, obtained from b. (**d**) Interpretive draw. C26b is interpreted as a partial hand-print of which only digit traces are preserved, interfering with a metatarsal trace deeply imprinted on a muddy, highly plastic, substrate.

After a walk of approximately 150 m from the original opening of the cave and a climb of about 12 m, the group arrived at the *‘Corridoio delle impronte’*. They proceeded roughly in single file, with the smallest individual behind, and walked very close to the side wall of the cave, a safer approach also used by other animals (e.g. Canidae *incertae sedis* and bears) when moving in a poorly lit and unknown environment. The slope of the tunnel floor, inclined by about 24°, may have further forced the individuals to proceed along the only flat area in the lower corridor, a couple of meters from the left wall of the cave. About 10 m from the *‘Corridoio delle impronte’*, the cave roof drops to below 80 cm and members of the group were forced to crawl (Figure 11B), placing their hands (Figure 12) and knees (Figure 8b) on the clay substrate (Figure 9) (see also Video 1).

**Video 1.** Virtual exploration of the cave showing the crawling locomotion adopted by the Palaeolithic group to cross the main gallery and to access the innermost rooms of the cave.

After a few meters, the group leader stopped, impressing two parallel calcigrade footprints, possibly to decide on the next movement and proceeded to cross the parts where the cave roof was at its lowest. The other individuals also stopped at the same place as the leader, and then proceeded along the same path by crawling and following the group leader, as indicated by the timing reconstructed from interactions between the tracks (Figure 9, d3).

After passing a bottleneck of blocks and stalagmites, the party descended for about ten meters along a steeply sloping surface. The whole group traversed a small pond, leaving deep tracks on the plastic waterlogged substrate, climbed a slope of 10 m beyond the ‘*Cimitero degli orsi’*, and finally arrived at the terminal room *‘Sala dei Misteri’*, where they stopped. On the walls, several charcoal traces, generated by the torches, are preserved.

Some charcoal handprints produced by a flexed reaching up more than 170 cm on the roof of the ‘*Sala dei Misteri’* confirm that the tallest individuals (Morphs. 4 and 5) were able to touch this part of the gallery. The fact that their footprints are not preserved relates to the loss of the central portion of the hall floor. In the same room, the adolescent and children started collecting clay from the floor and smeared it on a stalagmite at different levels according to height, as suggested by the breadth and relative distribution of the finger flutings on the karst structure. During their sojourn in the innermost room of the cave the young individual, that produced Morph. 2, imprinted ten clear heel traces (Citton et al., 2017), which are here interpreted as calcigrade tracks produced by a trackmaker who is momentarily standing-still to excavate and manipulate clay as was also recorded for the ‘Salle des Talons’ at Tuc d’Audoubert cave (Pastoors et al., 2015).

**Figure 13.**
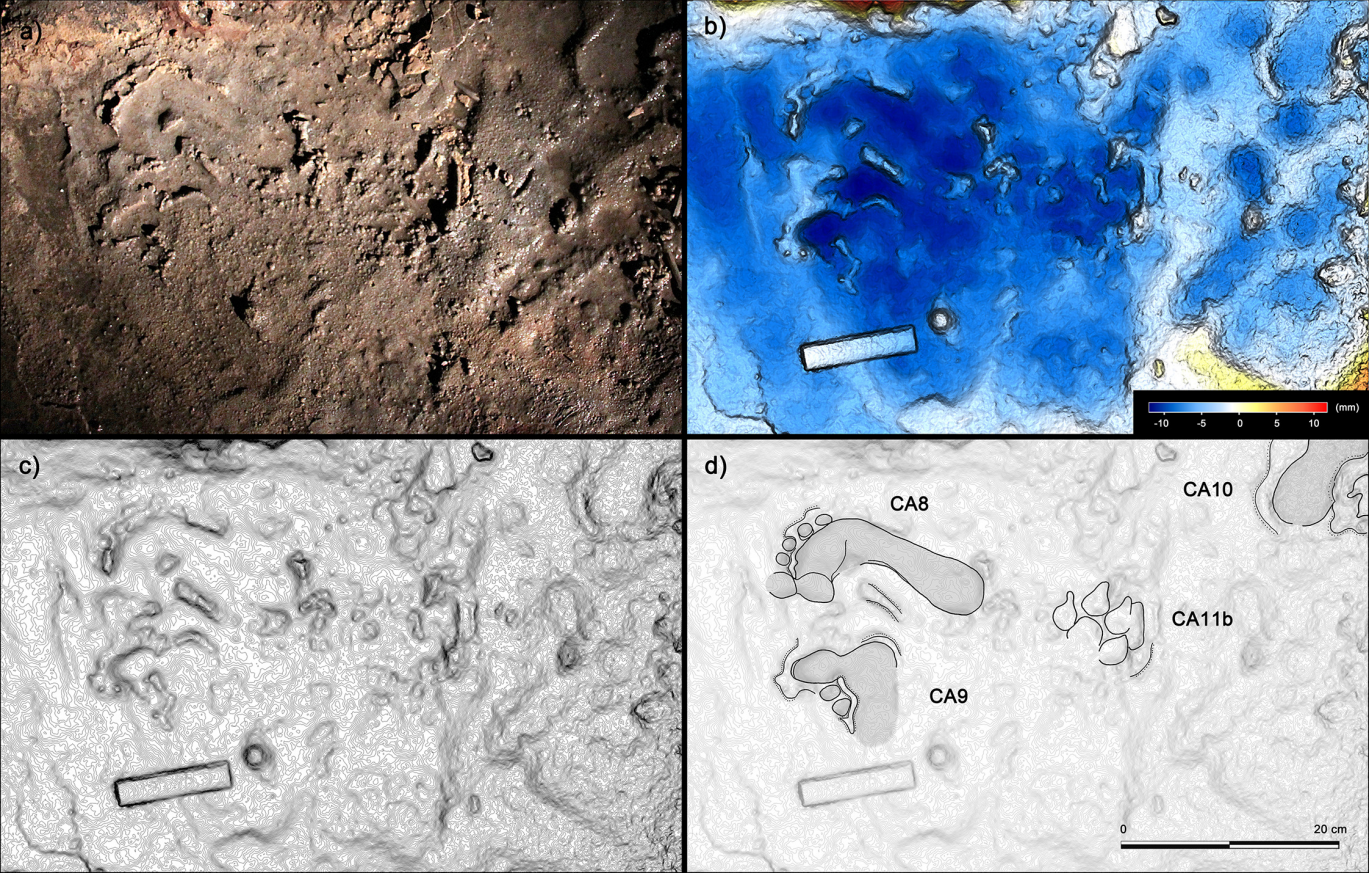
Shallow human tracks from the ‘upper corridor’. (**a**) Tracks CA8, CA9, CA10 and Ca11b from the sector C of the ‘upper corridor’ (see Figure 1 main text). (**b**) Digital terrain model obtained from high-resolution photogrammetry. (**c**) Topographic profile with contour lines, obtained from b. (**d**) Interpretive draw. Tracks were impressed on a hard carbonate substrate covered by a thin muddy deposit, few millimeters in thickness.

After stopping for several minutes (considering the quantity and ubiquity of the tracks), they exited and followed a route which did not always adhere to that followed on entry. After passing the small pond, they crossed the upper corridor following a more comfortable and safer route (Figure 11C). It is important to note that in the upper corridor all the prints point in the direction of the exit (Figures 11C and 13) while in the lower corridor, with the axis of the foot oriented parallel to the walls, most of the footprints are directed toward the interior of the cave.

### Concluding remarks

A holistic analysis comprising several lines of inter-related ichnological evidence enabled reconstruction of several snapshots depicting a small and heterogeneous group of Upper Palaeolithic people that explored a cave about 14,000 years ago (Video 1). They traversed the uneven topography of the cave floor and carried out social activities in the most remote room, leaving evidence in their traces of a unique testimony to human curiosity.

The lower corridor was traversed on entry into the cave and documents the first unequivocal evidence of crawling locomotion in the human ichnological record. This mode of locomotion was adopted by the explorers to obviate variation in the height of the cave roof. As no outgoing footprints were documented in the lower corridor it appears that the group chose to exit through the upper corridor, as the cave roof is higher and the substrate firmer. An additional reason for choosing this path of exit could have been the exploratory and curiosity factor to follow a different and unexplored path to reach the cave exit.

Anatomical features clearly registered on the substrate indicate that the lower limbs of the individuals were not covered with clothing. Our study also confirms that very young children actively participated in the activities of the Upper Palaeolithic populations, even in seemingly dangerous tasks, such as the deep exploration of the cave environment lit only with torches. As recently suggested for other European caves (Pastoors et al., 2015; Pastoors et al., 2017; Ledoux et al., 2017) the ‘Grotta della Bàsura’ site strongly supports the hypothesis that the cave exploration in Upper Paleolithic was carried out by groups of heterogeneous age and gender.

The Epigravettian necropolis of the Arene Candide Cave (AMS dates spanning 12,820–12,420 cal BP for the first phase and 12,030–11,180 cal BP for the second phase), consisted of a ‘mixed’ sample of people (males, females, adults, children) and suggests an Upper Palaeolithic people composition very similar to that highlighted in the ‘Grotta della Bàsura’ (Riel-Salvatore et al., 2018; Sparacello et al., 2018). The burial of a newborn infant, together with grave goods, recently discovered in the Arma di Veirana cave (Erli, Savona, Liguria) situated in a valley 10 km from the coast, further indicates that women and children systematically followed the movements of the group in the territory (F. N. pers. obs.) (Negrino et al., 2017) and shared, at least in part, the activities of men and had similar personal adornment. The tracks left in the Bàsura indicate that the behavior of hunter-gatherers was not always driven by subsistence requirements, but as manifested by many ethnographic examples, also by fun and frivolous activities.

## Materials and methods

### Chronology

Radiometric dating of charcoals previously established the presence of humans in the cave to the Upper Palaeolithic, around 12,340 ± 160 years BP (De Lumley and Giacobini, 1985). The stalagmite crust preserving the footprints and incorporating fragments of coal is dated to between 14,300 ± 800 and 13,100 ± 500 (Yokoyama et al., 1985). The final phase of stalagmite growth, which closed the entrance and sealed the ‘time capsule’, occurred at 12,000 ± 1100 (Yokoyama et al., 1985).

**Figure 14.**
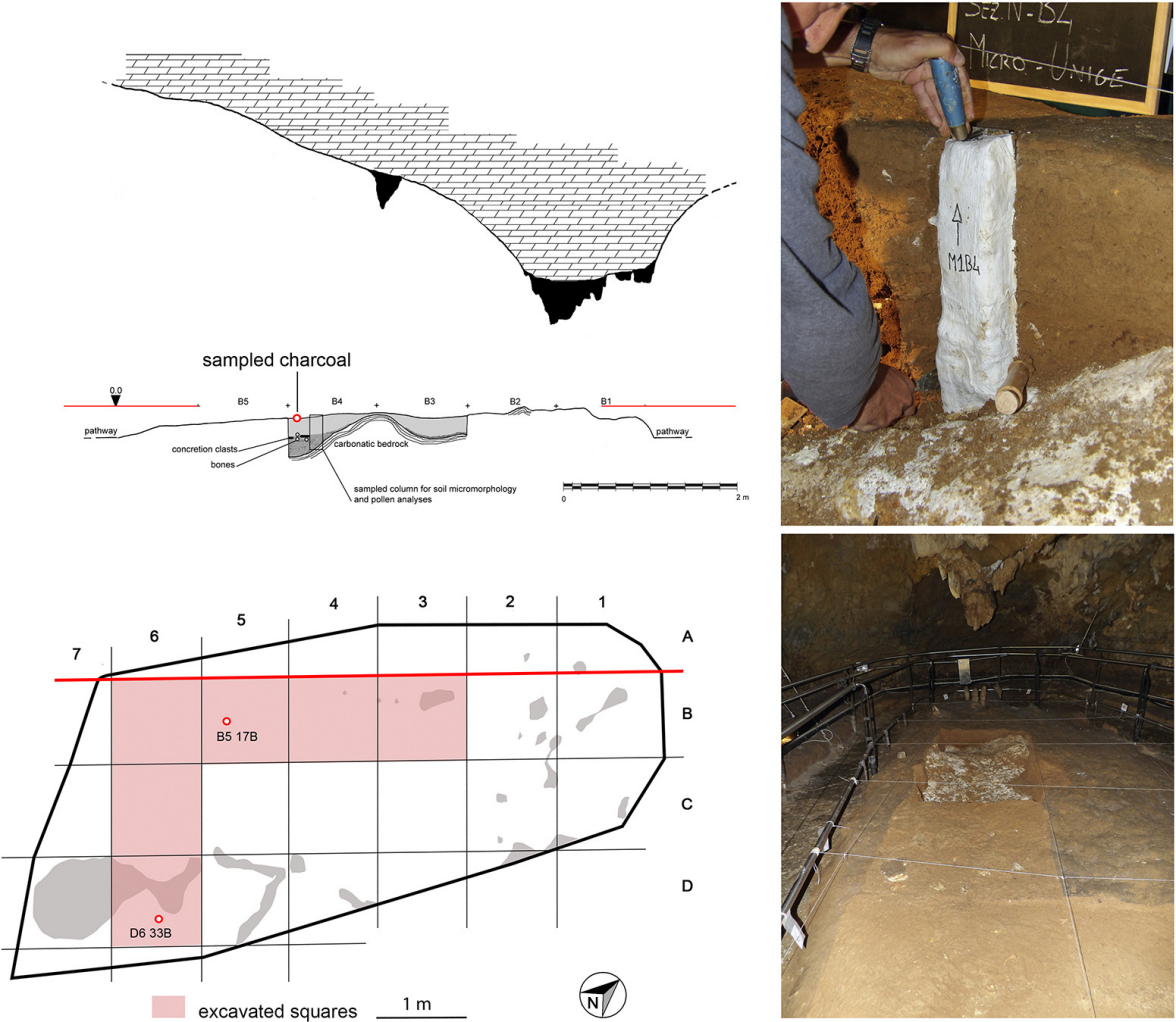
Profile and map of the archaeo-paleontological excavations in the Mysteries Hall (left), soil micromorphology sampling and view of the excavations (right). The sampled charcoal for dating are highlighted by a red dot.

New radiometric dating of charcoal samples of *Pinus* t. *sylvestris/mugo* was undertaken in 2017 at a AMS facility at Groningen (NL) on (Table 1). Material was collected from the trampled palaeosurface during recent excavations inside the ‘*Sala dei Misteri*’ (Figure 14).

### Laser scanning acquisition

Documentation of the sequence of events was contextualized and visualized on the rough cave topography through three-dimensional mapping of the cave performed by laser-scanning. The main landmarks of the cave were digitally recorded using the laser scanner ScanStation2 Leica and ScanStation C10 Leica. The scans were performed at 360° (acquisition grid of the point cloud of 2 × 2 cm a probe 7 m and in correspondence to the areas with the highest concentration of traces, an acquisition grid of 0.5 × 0.5 cm probe 7 m). In total, 23 stations were run (nine in the *‘Sala dei Misteri’* and 14 in the *‘Corridoio delle impronte’* areas); 38 targets (16 in the *‘Sala dei misteri’* and 22 in the *‘Corridoio delle impronte’*) were used for the point clouds registration. The Leica Geosystems HDS Cyclone 9.1 software was used to process the data. The recording shows a final alignment error of 2 mm for the model of the *‘Sala dei Misteri’* and 1 mm for the *‘Corridoio delle impronte’* (Video 1). From the models, reliefs were obtained at various degrees of detail that enabled georeferencing of all traces. The original cast performed in 1950 were digitally acquired via HDI Advance structured-light 3D Scanner R3x, with a resolution of 0.25 mm at 600 mm FOV (field of view). The data were processed with FlexScan3D Software (Figures 6, 7, 8d and 9d).

### Digital photogrammetry

Several photogrammetric models were obtained using several photos taken with 24 Megapixel Canon EOS 750D (18 mm focal length). The software used to build models is Agisoft PhotoScan Pro (www.agisoft.com). High-resolution Digital Photogrammetry is based on Structure from Motion (SfM) (Ullman, 1979) and Multi View Stereo (MVS) (Seitz et al., 2006) algorithms and produces high-quality dense point clouds. The accuracy of the obtained models is up to 1 mm for close-range photography. The reconstructed 3D surfaces were then processed in the open-source software Paraview. False colored models with contour lines, highlighting general morphology and differential depth of impression of the traces, were obtained (Figures 9a, 12 and 13).

### Analysis of human footprints

All recognized tracks (107 human traces) were analyzed directly in the field through a morphological approach using available landmarks (Robbins, 1985). The differential depth of each individual impression was analyzed directly in the field to infer the complex and multiphase biomechanics. All isolated footprints, and those associated with trackways, were drawn in the field on plastic film. All morphological and dimensional data collected in the field were double-checked by using photos and photogrammetric models.

In addition, the original casts of the footprints from the 1950s were also used and analyzed. Their positions in the cave were verified and it was established that all the footprints identified during the early explorations are still visible in situ (Figure 1, Table 4). Some of them have suffered alteration and loss of detail and others have been partially damaged. For this reason, in some instances, we have integrated morphometric data of the in situ footprints with those of plaster casts. In addition, the following two indices were considered: Footprint index (FI), equal to foot width/foot length x100 and Arch angle (Aa), represented by the angle between the footprint medial border line and the line that connect the most medial point of the footprint metatarsal region and the apex of the concavity of the arch of the footprint (Clarke, 1933). In the reconstruction of body dimensions and age, only the foot measurements derived from the better-preserved footprints were used (Table 3).

**Table 4.** Footprints and relative measures used for the Principal Component Analysis. Anatomical abbreviations as in Materials and methods Section.

Footprints			Lengths							Widths	
ID	IN SITU	CAST 1950–51	Dt1-BL	Dt2-BL	Dt3-BL	Ball medial (mtm-BL)	Ball lateral (mtl-BL)	Heel medial (ccm-BL)	Heel lateral (ctul-BL)	Ball (mtm-horiz)	Heel (ctul-horiz)
			(cm)	(cm)	(cm)	(cm)	(cm)	(cm)	(cm)	(cm)	(cm)
SM3	X		13	12.3		10.5	9.2	2.3	2.4	5.5	4.3
SM4	X		13.5	12.5	11.5	10.2	8	2.2	1.6	5.5	4.2
SM43	X		13.5	13.5		10.5	9.4	2.2	1.8	6	4.6
SM17	X		14.2	13.8	13.5	10.2	8.5	2	1.8	6.5	4
SM5	X		17	16.8	15.5	12	12	2.5	2	6.5	5
SM42	X		17	16.8		12.5	11.5	3.4	2.5	7	5.4
SM26	X		18	16.5	15			2	3		5
CA8	X	X	20.2	19.8	19.4	15	13	2.6	2	7.5	4.5
C10	X		20.5		19.2	15.5	12.5	2.8	2.6	7	5.6
SM15	X		20.5	20.5	18.5	15.4	13	4	2	7	6
SM11	X		21	20.2	19.7	15.4	14.5	3.3	2.3	7.5	6
SM6	X		21	21.5	20.5	17.5	15.5	4	2.2	9.2	5.7
SM1	X	X	21.2	21.3	20.8	17.4	14.8	3.8	2.5	8	6
C33	X	X	22.2	21	19.5	15.5	10.7	2.5	2	10	5.8
C36	X	X	22.7	21.7	19.5		14.5	3	2.5		5.5
M21		X		21.6	21.3	16.7	14.6	3.5	2.4	9.6	6.2
CA1	X	X	22.4	21.8	20.8	14.8	12.8	3.1		8	
CA2	X		22.5	22	21	17.3	15.2	3.5	3.5	8.8	6.4
C61	X	X	23	21.7	20.8	16.4	14	3.8	3	7.5	5.4
C63	X	X	23.3	22.2	20.8	17	14	3.5	3.8	8.5	5.3
C60	X	X	25.3	24.2	22.7	18	14.8	3.5	3.3	10.5	6.4
C37	X	X	25.7	23.8	22.5	18.4	14.7	3.7	3.5	10.5	7
C35B	X	X	26.2	24.8	22.8	17	13.7	3.7	2.7	9.5	6.7

### Principal Component Analysis

**Figure 15.**
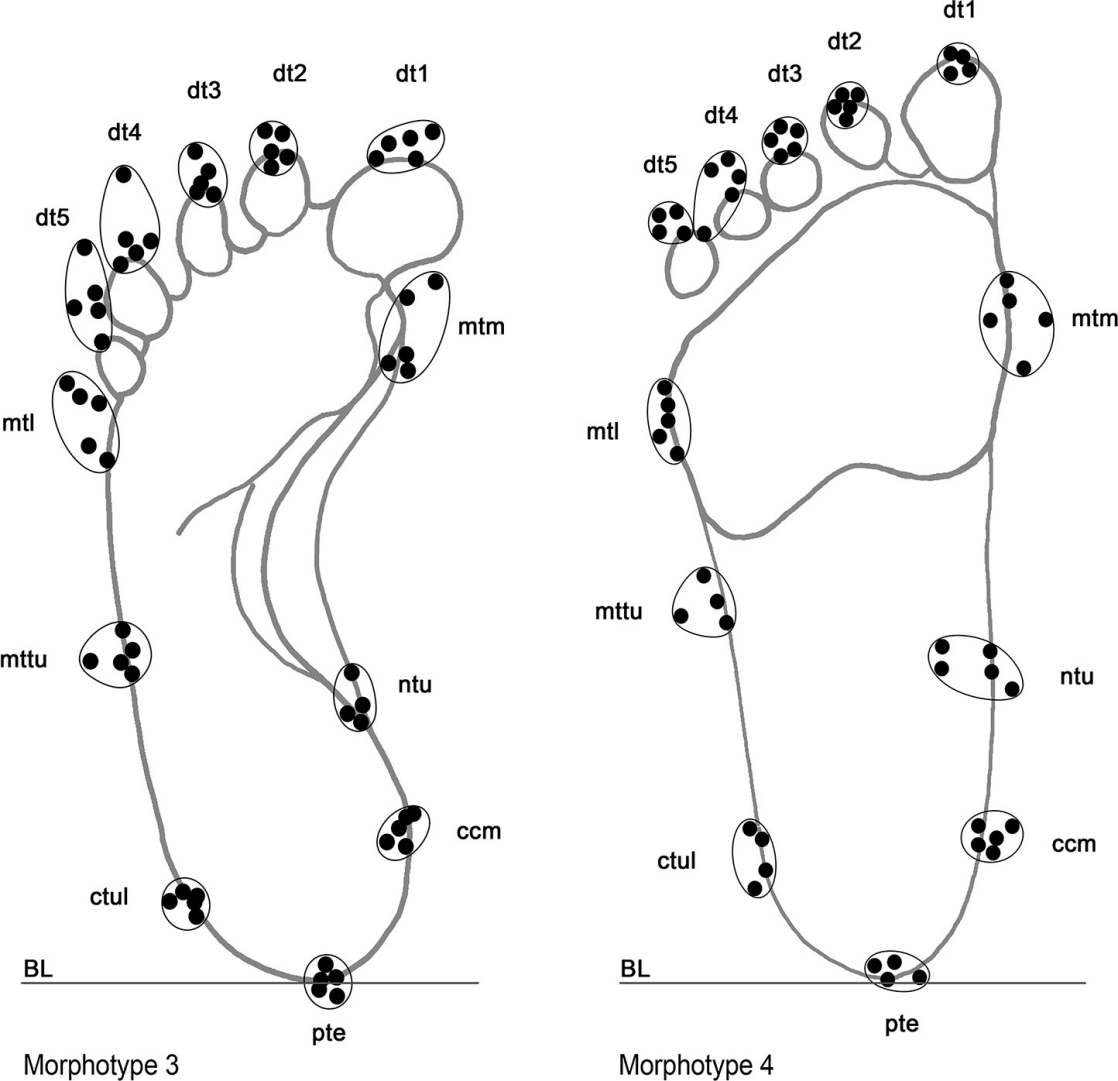
Adopted landmarks utilized to perform morphometric analysis, showed in two distinct morphotypes (Morphs 3 and 4) as example. Landmarks in the distal portion of digit traces 4, 5, and in the medial, central and lateral portions of the sole trace were not considered reliable enough for the large variability, higher than the fixed error value (±0.5 cm).

The 23 better preserved footprints were subjected to a Principal Component Analysis (PCA) using the software PAST 3.10 (Hammer et al., 2001). Homologous points were selected on the footprints for the measurements (Robbins, 1985) (Figure 15). These included nine anatomical lengths and widths (foot lengths (Dt1-BL, Dt2-BL, Dt3-BL); ball medial length (mtm-BL); ball lateral length (mtl-BL); heel medial length (ccm-BL); heel lateral length (ctul-BL); widths of ball (mtm-horiz.) and heel (ctul-horiz.) (Table 4). The raw data were log-transformed before the analysis to fit linear models and for the correspondence of the log transform to an isometric null hypothesis (Chinnery, 2004; Cheng et al., 2009; Romano and Citton, 2015; Romano and Citton, 2017; Romano, 2017). Missing entries were treated according to the ‘iterative imputation’ in PAST 3.10, preferable to the simple ‘mean value imputation’ (Hammer, 2013). The results of the PCA are reported in the scatter plots of Figure 5a, whereas the loadings for the first three principal components are provided as supplementary information (and Appendix 1 Tables A, B, Figure 5—figure supplement 1).

### Stature

It is possible to estimate stature from foot length (Robbins, 1985; Oberoi et al., 2006; Krishan and Sharma, 2007; Kanchan et al., 2008; Pawar and Pawar, 2012). Stature varies with race, age, sex, heredity, climate and nutritional status. Based on skeletal evidence, it is thought that the body proportions of terminal Upper Palaeolithic individuals was similar to that of modern humans (Trinkaus, 1997; Ruff et al., 2005; Shackelford, 2007) but the foot length/stature ratio was considered highly uncertain, between 0.15 and 0.16. Consequently, we calculated the foot length/stature ratio based on a sample of terminal Upper Palaeolithic adult individuals (n.8) from the Italian Peninsula (Corrain, 1977; Paoli et al., 1980; Formicola et al., 1990; Mallegni and Fabbri, 1995; Mallegni et al., 2000). The calculated ratio is found to be 0.1541, which is close to those proposed for modern humans between the XIX and XX centuries (Robbins, 1985; Topinard, 1878). Stature estimation from the length of long bones is commonly used in forensic medicine. In this study, we used the percutaneous length of the tibia to check the stature of the Morph. 5 as this measurement is known to have a strong correlation with body height. We used the relation S = 101.85 + 1.81 x PCTL ±3.73 for male and S = 77.86 + 2.36 x PCTL ±2.94 for female (where S = stature and PCTL = Percutaneous tibial length) (Lemtur et al., 2017). For a tibial length of 35 cm, the stature of Morph. 5 is 165.2 ± 3.73 cm, which is comparable to the stature assumed from foot length (166.99 ± 2.93 cm).

### Body mass

Body mass estimates were derived from footprint parameters, based on the assumption that human body proportions have been constant through time (Dingwall et al., 2013). Regression formulae are based on mature individuals ranging between 154 and 185 cm in stature (weight Kg = 4.71 + (1.82xFL)) (Dingwall et al., 2013; Bavdekar et al., 2006; Ashton et al., 2014) or on children (Grivas et al., 2008) with an average height of 147,44 cm (weigth Kg = −71.142 + (5.259xrigthFL). We have used these formulae for the individual taller than 147 cm (Table 3), stature: (b) (Bavdekar et al., 2006), (c) (Grivas et al., 2008). For the three smaller individuals, we used formulae based on extant Caucasian children between 6 and 11 years old (n. 7147) ranging in stature between 118.6 and 145.7 cm (Malina et al., 1973) to develop a mathematical relation between foot length and body mass for young individuals. The report is nonlinear and expressed by the formula mass = 2.2897 e^0.126FL^ (Citton et al., 2017). No dataset are available for the smaller individual. We have therefore hypothesized the body mass of MP1 by the trend-line derived from previous formulae.

### Age

Foot length varies according to age and gender. Studies on the relationship size/age of the foot in extant juvenile individuals (Fryar et al., 2012; Müller et al., 2012) have highlighted that 1-year-old individuals have a foot length equal to 13.07 ± 1.59 cm, reaching 24.4 ± 2.96 cm at the age of 13 years. The age estimation based on growth curves built on extant populations is very similar. However, we must bear in mind that the reference anthropometric data mainly refer to modern well-nourished populations, with a body mass most likely higher at the same age. Anthropometric studies suggest that the morphology of the foot changes and becomes more elongated when the arch stabilizes around the age of six (Müller et al., 2012). In extant human populations, from the fifth or sixth years of age, arch angles vary from 21° (3–4 years) to 43° (9–11 years) in young males, and from 26° (3–4 years) to 47° (9–11 years) in young females (Forriol and Pascual, 1990). As a result, morphotypes 2 and 3 seem to be similar, likely suggesting a corresponding similarity of age between producers of the two morphotypes. Growth curves based on extant populations with an average height similar of those of the Late Upper Paleolithic provided an estimate of the age of the trackmakers. For MP5, the wide and stout morphology is here interpreted as an adult stage with the partial collapse of the plantar arch.

### Gender

Sex determination established from the foot has been proposed using Foot index and threshold values. However, this approach is not entirely accepted and some researchers pointed out that the threshold value could vary significantly between populations, thus making it very speculative that gender estimations could be determined from foot morphology (Walia et al., 2016). These variations could be due to fact that anatomic structures of the foot manifest ethnic and regional variations owing to climatic factors, physical activities, socio-economic status and nutritional conditions. Despite the method, uncertain arch angle and footprint morphology suggests a possible male as trackmaker of the largest footprint group. No definitive gender can be inferred for Morphotypes 1, 2, 3, 4.

## Data Availability

All the data are included in tables within the main text.

## Additional files

Supplementary file 1. Supplementary table with all the tracks analyzed in the study.

Transparent reporting form

## Appendix 1

10.7554/eLife.45204.026

**Appendix 1—table 1.** Scores obtained from the Principal Component Analysis.

ID	PC 1	PC 2	PC 3	PC 4	PC 5	PC 6	PC 7	PC 8	PC 9
SM3	−0.39913	0.10426	−0.064014	0.014386	−0.025072	0.016054	0.016716	0.011934	0.0074328
SM4	−0.50068	−0.042109	−0.014907	0.032287	0.0032614	−0.029028	0.019643	−0.026998	0.0033618
SM43	−0.40104	−0.019701	−0.0054895	0.018735	0.0016977	0.027812	−0.0033284	0.0083841	−0.0075861
SM17	−0.42507	−0.0052715	0.061746	0.0063252	−0.038382	−0.016919	−0.0164	0.0059965	−0.0012712
SM5	−0.1976	−0.035523	−0.0043672	−0.03242	0.024328	0.032498	−0.025789	−0.011295	−0.0026746
SM42	−0.086943	0.013761	−0.08784	0.050378	−0.009608	−0.0043334	−0.025838	0.015148	0.0033864
SM26	−0.20626	0.16623	0.063691	−0.021075	0.045203	0.0078808	−6,97E-02	−0.013948	−0.00040047
CA8	−0.051293	−0.079409	0.054639	−0.097538	−0.026619	−0.014175	−0.002064	0.014186	0.00038865
C10	0.0058167	0.019522	0.0058477	−0.035311	0.03647	−0.00018801	0.032056	0.019886	0.0034661
SM15	0.040758	−0.12904	−0.095157	0.017228	0.043206	−0.034662	0.00082729	−0.005932	−0.0085001
SM11	0.06668	−0.064129	−0.032745	−0.023447	0.034482	0.026778	−0.0049565	0.0063519	0.0068743
SM6	0.15624	−0.12859	−0.045021	0.0020611	−0.050337	0.019214	0.001418	−0.0080637	−0.0071906
SM1	0.14791	−0.061674	−0.059992	−0.0097849	0.00034344	0.0088016	0.019386	0.010087	−0.0078662
C33	0.0099173	−0.09178	0.16838	0.043167	0.0021726	−0.017624	0.012848	0.010468	−0.0049062
C36	0.094929	−0.029812	0.032258	−0.047049	0.0025717	0.013716	−0.009842	−0.02199	0.0015418
M21	0.16838	−0.0796	0.021892	0.019189	−0.011351	0.022302	−0.0073946	0.0015577	0.014584
CA1	0.16391	0.21771	−0.0025937	0.0040729	0.0067048	−0.016137	−0.017977	0.012136	−0.0084853
CA2	0.22089	0.072731	−0.022013	0.006969	−0.0012392	0.036927	0.012619	−0.0038898	−0.0063643
C61	0.15271	0.023445	−0.066418	−0.034022	−0.0050802	−0.045788	−0.0011026	−0.0017506	0.007746
C63	0.19883	0.1176	−0.020327	−0.025171	−0.043065	−0.029182	0.0070871	−0.013733	−0.00144
C60	0.28381	0.030453	0.049268	0.0231	−0.021083	0.01058	−0.0018458	−0.0051978	−0.00067736
C37	0.3109	0.046052	0.029704	0.054401	−0.0019844	0.016719	0.011316	−0.0085163	0.0054307
C35B	0.24634	−0.045122	0.033459	0.033518	0.033381	−0.031244	−0.01731	0.0051775	0.0031501

**Appendix 1—table 2.** Loadings for each principal components. a, Dt1-BL; b, Dt2-BL; c, Dt3-BL; d, Ball medial (mtm-BL); e, Ball lateral (mtl-BL); f, Heel medial (ccm-BL); g, Heel lateral (ctul-BL); h, Ball (mtm-horiz); i, Heel (ctul-horiz). Anatomical abbreviations as in Methods section.

	PC 1	PC 2	PC 3	PC 4	PC 5	PC 6	PC 7	PC 8	PC 9
a	0.36632	−0.031724	0.28459	−0.15437	0.31322	−0.38384	−0.061146	−0.3656	0.61412
b	0.36721	−0.086634	0.19468	−0.1893	0.19705	−0.27853	−0.3069	−0.13269	−0.74546
c	0.34834	−0.062518	0.10263	−0.17626	−0.035551	−0.093333	−0.12422	0.88253	0.16995
d	0.34521	−0.15	0.03307	−0.16341	−0.13051	−0.019176	0.88858	−0.036893	−0.14898
e	0.33106	−0.13532	−0.24619	−0.50577	−0.065819	0.67412	−0.2218	−0.19439	0.099588
f	0.35243	−0.24194	−0.73379	0.3425	−0.19673	−0.31842	−0.1176	−0.070489	0.051056
g	0.32186	0.93175	−0.1145	0.050472	−0.09853	0.019641	0.025317	−0.030012	−0.030921
h	0.30501	−0.13213	0.50725	0.46978	−0.57082	0.21548	−0.15222	−0.12269	0.031883
i	0.245	−0.051134	−0.0026745	0.53499	0.68278	0.40417	0.10113	0.098963	−0.039702

## References

[bib1] Ashton N, Lewis SG, De Groote I, Duffy SM, Bates M, Bates R, Hoare P, Lewis M, Parfitt SA, Peglar S, Williams C, Stringer C (2014). Hominin footprints from early pleistocene deposits at Happisburgh, UK. PLOS ONE.

[bib2] Ast H (2001). Kienspan Und Unschlitt, beleuchtungimländlichenalltag. Kulturbeilage Zum Amtsblatt Der Bezirkshauptmannschaft Wiener Neustadt.

[bib3] Bavdekar SB, Sathe S, Jani P (2006). Prediction of weight of indian children aged upto two years based on foot-length: implications for emergency areas. Indian Pediatrics.

[bib4] Blanc AC (1960). Le palline d’argilla della Grotta della Bàsura. Rivista Di Studi Liguri.

[bib5] Blanc AC, Pales L, Lamboglia N (1960). Le Vestigia Umane Nella Grotta Della Bàsura a Toirano.

[bib6] Cheng Y-N, Holmes R, Wu X-C, Alfonso N (2009). Sexual dimorphism and life history of *Keichousaurus hui* (Reptilia: Sauropterygia). Journal of Vertebrate Paleontology.

[bib7] Chiappella VG (1952). Orsi e uomini preistorici nella grotta della strega. Rivista Del Comune.

[bib8] Chinnery B (2004). Morphometric analysis of evolutionary trends in the ceratopsian postcranial skeleton. Journal of Vertebrate Paleontology.

[bib9] Citton P, Romano M, Salvador I, Avanzini M (2017). Reviewing the upper pleistocene human footprints from the ‘Sala dei Misteri’ in the Grotta della Bàsura (Toirano, northern Italy) cave: An integrated morphometric and morpho-classificatory approach. Quaternary Science Reviews.

[bib10] Clarke HH (1933). An objective method of measuring the height of the longitudinal arch in the foot examinations. Research Quarterly. American Physical Education Association.

[bib11] Corrain C (1977). I resti scheletrici della sepoltura epigravettiana del “Riparo Tagliente’’ in Valpantena (Verona). Bollettino Del Museo Civico Di Storia Naturale Di Verona.

[bib12] De Lumley MA, Giacobini G (1985). Le impronte di piedi umani. Rivista Di Studi Liguri.

[bib13] de Lumley H, Vicino G (1984). New data concerning the dating and interpretation of human footprints present in the “Grotta della Basura” at Toirano (Savona, Northern Italy). Results of an international round table. Journal of Human Evolution.

[bib14] Dingwall HL, Hatala KG, Wunderlich RE, Richmond BG (2013). Hominin stature, body mass, and walking speed estimates based on 1.5 million-year-old fossil footprints at Ileret, Kenya. Journal of Human Evolution.

[bib15] Formicola V, Frayer DW, Heller JA (1990). Bilateral absence of the lesser trochanter in a late epigravettian skeleton from arene candide (Italy). American Journal of Physical Anthropology.

[bib16] Forriol F, Pascual J (1990). Footprint analysis between three and seventeen years of age. Foot & Ankle.

[bib17] Fryar CD, Gu Q, Ogden CL (2012). Anthropometric reference data for children and adults: United States, 2007-2010. Vital and Health Statistics. Series 11, Data From the National Health Survey.

[bib18] Giacobini G, Arobba D, Maggi R, Vicino G (2008). *La Grotta della Bàsura e il "mito neandertaliano"*. Toirano E La Grotta Della Bàsura. Conoscere, Conservare E Gestire Il Patrimonio Archeologico E Paleontologico.

[bib19] Giannotti S, Arobba D, Maggi R, Vicino G (2008). La grotta della Bàsura (Toirano): rilettura e aggiornamento dei dati archeologici. Toirano E La Grotta Della Bàsura. Conoscere, Conservare E Gestire Il Patrimonio Archeologico E Paleontologico.

[bib20] Grabner M, Klein A, Geihofer D, Reschreiter H, Barth FE, Sormaz T, Wimmer R (2007). Bronze age dating of timber from the salt-mine at Hallstatt, Austria. Dendrochronologia.

[bib21] Grabner M, Klein A, Reschreiter H, Barth F-E, Anreiter P (2010). In Mining in European History and Its Impact on Environment and Human Societies – Proceedings for the 1st Mining in European History-Conference of the SFB-HIMAT.

[bib22] Grivas TB, Mihas C, Arapaki A, Vasiliadis E (2008). Correlation of foot length with height and weight in school age children. Journal of Forensic and Legal Medicine.

[bib23] Hammer Ø, Harper DAT, Ryan PD (2001). PAST: paleontological statistics software package for education and data analysis. Palaeontologia Electronica.

[bib24] Hammer Ø (2013). PAST Paleontological Statistics Version 3.0: Reference Manual.

[bib25] Janssens L, Giemsch L, Schmitz R, Street M, Van Dongen S, Crombé P (2018). A new look at an old dog: bonn-oberkassel reconsidered. Journal of Archaeological Science.

[bib26] Kanchan T, Menezes RG, Moudgil R, Kaur R, Kotian MS, Garg RK (2008). Stature estimation from foot dimensions. Forensic Science International.

[bib27] Krishan K, Sharma A (2007). Estimation of stature from dimensions of hands and feet in a north indian population. Journal of Forensic and Legal Medicine.

[bib28] Lamboglia N (1960). Le vestigia umane Nella Grotta della bàsura a toirano. Rivista Di Studi Liguri.

[bib29] Ledoux L, Fourment N, Maksud F, Delluc M, Costamagno S, Goutas N, Klaric L, Laroulandie V, Salomon H, Jaubert J (2017). Traces of human and animal activity (TrAcs) in Cussac cave (Le Buisson-de-Cadouin, Dordogne, France): Preliminary results and perspectives. Quaternary International.

[bib30] Lemtur M, Rajlakshmi C, Damayanti D (2017). Estimation of stature from percutaneous length of ulna and tibia in medical students of Nagaland. JDMS.

[bib31] Lupo KD (2017). When and where do dogs improve hunting productivity? the empirical record and some implications for early upper paleolithic prey acquisition. Journal of Anthropological Archaeology.

[bib32] Maineri D (1985). La scoperta della grotta della bàsura a toirano. Atti della tavola rotonda “La Grotta preistorica della Bàsura”, Toirano, 11-13 novembre 1983. Rivista Di Studi Liguri.

[bib33] Malina RM, Hamill PVV, Johston FE, Lemeshow S (1973). Selected body measurements of children 6-11 years: United States. Vital Health Stat.

[bib34] Mallegni F, Bertoldi F, Manolis S (2000). Palaeobiology of two gravettian skeletons from veneri cave (Parabita, Puglia, Italy). Homo : Internationale Zeitschrift FüR Die Vergleichende Forschung Am Menschen.

[bib35] Mallegni F, Fabbri PF (1995). The human skeletal remains from the upper palaeolithic burials found in Romito cave (Papasidero, Cosenza, Italy). Bulletins Et Mémoires De La Société d'anthropologie De Paris.

[bib36] Molleson TI, Oakley KP, Vogel JC (1972). The antiquity of the human footprints of tana della basura. Journal of Human Evolution.

[bib37] Molleson T (1985). The antiquity of human footprints of tana della bàsura. Atti della tavola rotonda "La grotta preistorica della Bàsura, Toirano 11-13 novembre 1983. Rivista Di Studi Liguri.

[bib38] Morey DF, Jeger R (2015). Paleolithic dogs: why sustained domestication then?. Journal of Archaeological Science: Reports.

[bib39] Müller S, Carlsohn A, Müller J, Baur H, Mayer F (2012). Static and dynamic foot characteristics in children aged 1-13 years: a cross-sectional study. Gait & Posture.

[bib40] Negrino F, Benazzi S, Hodgkins J, Miller C, Orr C, Peresani M, Riel-Salvatore J, Strait D, Gravel-Miguel C, De Santis H, Leger E, Martini S, Perroni E, Laliberté A, Pothier Bouchard G, Starnini E, Zerboni A (2017). On-going research and first data from middle and upper palaeolithic sites of Liguria region. Bulletin Du Musée d'Anthropologie Préhistorique De Monaco.

[bib41] Oberoi DV, Kuruvilla A, Saralaya KM, Rajeev A, Ashok B, Nagesh KR, Rao NG (2006). Estimation of stature and sex from footprint length using regression formulae and standard footprint length formulae respectively. Journal of Punjab Academy of Forensic Medicine & Toxicology.

[bib42] Pales L (1960). Le vestigia umane nella grotta della Bàsura a Toirano. Rivista di Studi Liguri.

[bib43] Paoli G, Parenti R, Sergi S (1980). Gli scheletri mesolitici della caverna delle arene candide (Liguria). Memorie dell'Istituto Italiano Di Paleontologia Umana Roma.

[bib44] Pastoors A, Lenssen-Erz T, Ciqae T, Kxunta U, Thao T, Bégouën R, Biesele M, Clottes J (2015). Tracking in caves: experience based reading of pleistocene human footprints in french caves. Cambridge Archaeological Journal.

[bib45] Pastoors A, Lenssen-Erz T, Breuckmann B, Ciqae T, Kxunta U, Rieke-Zapp D, Thao T (2017). Experience based reading of pleistocene human footprints in Pech-Merle. Quaternary International.

[bib46] Pawar RM, Pawar MN (2012). "Foot length--A functional parameter for assessment of height". The Foot.

[bib47] Perri A (2016). A wolf in dog's clothing: Initial dog domestication and Pleistocene wolf variation. Journal of Archaeological Science.

[bib48] Riel-Salvatore J, Gravel-Miguel C, Maggi R, Martino G, Rossi S, Sparacello VS, Borgia V, Cristiani E (2018). New Insight into the Paleolithic Chronology and Funerary Ritual of Cavena delle Arene Candide. Palaeolithic Italy – Advanced Studies on Early Human Adaptations in the Apennine Peninsula.

[bib49] Robbins LM, Charles C (1985). Footprints. Collection, Analysis, and Interpretation.

[bib50] Romano M (2017). Long bone scaling of caseid synapsids: a combined morphometric and cladistic approach. Lethaia.

[bib51] Romano M, Citton P (2015). Reliability of digit length impression as a character of tetrapod ichnotaxobase: considerations from the Carboniferous-Permian ichnogenus *Ichniotherium*. Geological Journal.

[bib52] Romano M, Citton P (2017). Crouching theropod at the seaside. Matching footprints with metatarsal impressions and theropod authopods: a morphometric approach. Geological Magazine.

[bib53] Ruff C, Niskanen M, Junno JA, Jamison P (2005). Body mass prediction from stature and bi-iliac breadth in two high latitude populations, with application to earlier higher latitude humans. Journal of Human Evolution.

[bib54] Seitz SM, Curless B, Diebel J, Scharstein D, Szeliski R (2006). A comparison and evaluation of multi-view stereo reconstruction algorithms *CVPR* 2006.

[bib55] Shackelford LL (2007). Regional variation in the postcranial robusticity of late upper paleolithic humans. American Journal of Physical Anthropology.

[bib56] Sparacello VS, Rossi S, Pettitt P, Roberts C, Riel-Salvatore J, Formicola V (2018). New insights on final epigravettian funerary behavior at arene candide cave (Western Liguria, Italy). Journal of Anthropological Sciences = Rivista Di Antropologia : JASS.

[bib57] Théry-Parisot I, Thiébault S, Delannoy J-J, Ferrier C, Feruglio V, Fritz C, Gely B, Guibert P, Monney J, Tosello G, Clottes J, Geneste J-M (2018). Illuminating the cave, drawing in black: wood charcoal analysis at Chauvet-Pont d'arc. Antiquity.

[bib58] Tongiorgi E, Lamboglia N (1954). La Grotta Di Toirano.

[bib59] Topinard P (1878). Anthropology.

[bib60] Trinkaus E (1997). Appendicular robusticity and the paleobiology of modern human emergence. PNAS.

[bib61] Ullman S (1979). The interpretation of structure from motion. Proceedings of the Royal Society of London. Series B, Biological Sciences.

[bib62] Villotte S, Samsel M, Sparacello V (2017). The paleobiology of two adult skeletons from baousso da Torre (Bausu da ture) (Liguria, Italy): Implications for gravettian lifestyle. Comptes Rendus Palevol.

[bib63] Walia S, Modi BS, Puri N, Professor, Department of Anatomy, MMIMSR, Mullana, Ambala, Haryana, India (2016). Sexual dimorphism from foot dimensions and foot prints in haryanvi jat population. International Journal of Anatomy and Research.

[bib64] Yokoyama Y, Guanjun S, Nguyen Van H (1985). Dating of stalagmitic carbonates and bones of the bàsura cave at Toirano (Liguria, Italy) by the U-Th and U-Pa methods using alpha-and gamma-ray spectrometries. Rivista Di Studi Liguri.

